# Targeting T cell metabolism and polarization to modulate post-stroke immune responses and improve outcomes

**DOI:** 10.3389/fimmu.2026.1703552

**Published:** 2026-05-08

**Authors:** Juliane Gellrich, Nora Bödecker, Imke Reich, Johanna Ruhnau, Stefan Groß, Susanne H. Kirsch, Rolf Müller, Juliane Schulze, Antje Vogelgesang

**Affiliations:** 1 Department of Neurology, University Medicine, Greifswald, Germany; 2 Department of Inner Medicine B, University Medicine, Greifswald, Germany; 3 Helmholtz Institute for Pharmaceutical Research Saarland (HIPS), Helmholtz Centre for Infection Research (HZI), Saarland University, Saarbrücken, Germany; 4 German Centre for Infection Research (DZIF), Partner Site Hannover-Braunschweig, Braunschweig, Germany; 5 Department of Pharmacy, Saarland University, Saarbrücken, Germany

**Keywords:** antigen-specific, GFP, MCAO, Nur77, Soraphen A, stroke, T cell activation, Th17/Treg balance

## Abstract

**Background:**

T cells drive post-stroke secondary brain injury, with the Th17/Treg balance shaping post-stroke inflammation. Soraphen A (SorA) inhibits Th17 polarization while preserving Tregs. We examined SorA’s effects on post-stroke T cell activation – distinguishing antigen-specific from bystander activation – including inflammatory conditions induced by LPS.

**Methods:**

Male Nur77^GFP^ mice (12–14 weeks) underwent tMCAO. At reperfusion, mice received intraperitoneal LPS or vehicle; SorA or vehicle was given 2 h later and daily thereafter. MRI at 16 h and 7 d confirmed infarcts and measured lesion volumes. Functional outcomes were assessed by daily scoring and behavioral tests before surgery and at 2 and 6 d. T cell activation and polarization were analyzed by flow cytometry in brain, lungs, spleen, blood, and lymph nodes at 16 h, 2, 3, and 7 d, with GFP indicating antigen-specific activation.

**Results:**

LPS worsened functional outcomes and increased peripheral T cell activation post-stroke. SorA improved functional recovery, reduced peripheral T cell activation and enhanced antigen-specific T cell activation. SorA increased Treg in LPS-treated mice and reversed LPS-induced alterations in both T cell activation and behavior.

**Conclusions:**

Soraphen A modulates systemic and organ-specific T cell responses in experimental ischemic stroke and improves outcomes. SorA increases regulatory T cells, reverses LPS-associated T cell activation (CD25, CD69, PD-1) and behavioral deficits, and shifts responses toward antigen-specific T cell receptor-mediated activation. These results support SorA as a promising immunomodulatory strategy to refine post-stroke immunity, extend the therapeutic window, and improve outcomes with potential benefits in the context of post-stroke infections.

## 1 Introduction

The post-stroke immune response is strongly dependent on timing and localization. In the brain, a proinflammatory reaction occurs as peripheral immune cells, including T lymphocytes, infiltrate the tissue and worsen ischemic damage (1–3). T lymphocytes exacerbate the initial tissue damage caused by ischemia (4–6): Adoptive transfer of T cells to lymphocyte-deficient mice drastically increased the infarct volume (5, 6) while blocking T cell infiltration into the brain reduced the lesion size (7, 8). In contrast, the peripheral immune system is in a suppressed state, characterized by lymphopenia, atrophy of secondary lymphatic organs and immune cell dysfunction (9–14). Peripheral immunosuppression contributes to the development of post-stroke infections, that occur in about 30% of stroke patients and significantly aggravate outcome (10, 13, 15–17). As current causal interventions for ischemic stroke are all aiming at early blood flow restoration, only a small fraction of stroke patients can benefit (18, 19). Complementary therapies addressing alternative aspects of stroke pathology may help extend the therapeutic window into the subacute recovery phase. Among these, immunomodulatory strategies targeting the T cell response show particular promise.

Activated T cells can differentiate into various subsets, including pro-inflammatory Th17 cells and regulatory T cells (Treg). Stroke patients show an increased Th17/Treg ratio, which is a critical determinant for the extent of post-stroke inflammation and tissue damage (20–22). Elevated Th17 cell counts and IL-17A levels in the brain and blood correlate with stroke severity, poor outcomes, and cognitive decline, and are linked to blood-brain barrier damage and neuronal injury (20–31). The role of regulatory T cells has been intensively studied, yet remains controversial: The depletion of regulatory T cells has produced mixed results (32, 33) and while the amplification of Treg numbers led to decreased infarct volumes and improved functional outcome in a number of studies (21, 28, 34–37), some results indicate that Treg might worsen stroke pathology by promoting thrombus formation (32, 38). Treatment timing likely contributes to these conflicting findings.

T cell polarization depends on distinct metabolic conditions that can be targeted to push T cell polarization in a certain direction. In the Th17/Treg balance, regulatory T cells favor lipid oxidation and exogenous fatty acid uptake, while Th17 cells rely on *de novo* fatty acid synthesis (39–42). We utilized the myxobacterial natural product Soraphen A (SorA), an inhibitor of Acetyl Coenzyme A Carboxylase (ACC) 1 and 2 (43–45). ACCs catalyze the carboxylation of acetyl-CoA to malonyl-CoA, which is a key substrate for fatty acid synthesis. By blocking this pathway, SorA selectively impairs Th17 polarization while preserving Treg metabolism. SorA has proven beneficial in the EAE (experimental autoimmune encephalitis) mouse model of multiple sclerosis (40) and reduced the infarct volume in murine stroke (46). In our study, we expand the investigation of SorA’s impact on post-stroke T cell activation and its potential therapeutic benefit in the context of post-stroke infection significantly: 1.) To model systemic inflammation associated with post stroke infections, a common complication seen in stroke patients, we used intraperitoneal LPS injection—a well-established endotoxemia model known to induce systemic and neuroinflammation, as well as cognitive impairment (47–50). 2.) We characterize T cell subsets of major importance in stroke recovery, including activated T cells (characterized by expression of activation markers CD25, CD69 and/or PD-1) as well as Th17 and regulatory T cell subsets (characterized by expression of transcription factors RORγt or FoxP3, respectively). 3.) We take advantage of a transgene allowing us to identify and report the activation mode of T cells.

T cells can be activated either antigen-specifically via their T cell receptor (TcR) or antigen-independently through inflammatory signals. In the first three days after stroke, antigen-independent activation appears crucial, as T cell depletion shows protective effects within 24 hours of MCAO, and lesion size reduction is reversed by transferring T cells responsive to irrelevant antigens (4, 51, 52). However, antigen-specific T cells have been detected in the brain early after stroke (53) with antigen-dependent activation increasing in later stages. Clonal expansion was first observed on day 7 after MCAO, matching the typical 3–7 day antigen recognition period (54, 55). A narrowed TCR repertoire in brain-infiltrating Tregs also suggests antigen-specific activation (56). In stroke patients, T cell responses to neuronal antigens link to better outcomes, while reactivity to myelin antigens correlates with worse prognosis (57–59). Notably, inducing nasal or oral tolerance to myelin antigens reduces infarct size (60, 61). Targeting antigen-specific T cells may offer therapeutic potential, but further insights into post-stroke T cell activation, timing, and localization are needed.

We employ the Nur77^GFP^ transgenic mouse model to distinguish antigen-specific from antigen-independent T cell activation within a physiological TCR repertoire. Nur77 is an immediate early gene selectively induced by antigen receptor signaling in lymphocytes, but not by proinflammatory stimuli alone.

In the search for targeted immunomodulatory interventions, we investigated the effects of SorA on post-stroke antigen-specific T cell activation, the balance between pro-inflammatory and regulatory T cell subsets and on the increased inflammation associated with post-stroke infections.

## 2 Methods

### 2.1 Animals and housing

All animal experiments and the calculated sample sizes were approved by the local government authorities (Landesamt für Landwirtschaft, Lebensmittelsicherheit und Fischerei (LALLF) Mecklenburg-Vorpommern, 7221.3-1.1-040/17) and comply with the ARRIVE guidelines as well as the EU Directive 2010/63/EU for animal experiments.

Mice were bred locally at the Central Service and Research Facility of the University Medicine Greifswald. We used 12- to 14-week-old male Nur77^GFP^ mice to exclude variations due to estrous cycle influences. The Nur77^GFP^ mouse model was generated by Kristin Hogquist (C57BL/6-Tg(Nr4a1-EGFP/cre)820Khog/J mice, Stock No: 016617, The Jackson Laboratory) (62) and express eGFP under the control of the Nr4a1 promoter/enhancer regions. Nr4a1 encodes the immediate early gene Nur77, which is expressed after T cell activation via antigen receptor stimulation but not by inflammatory stimuli alone (62).

Two weeks before the experiments, mice were transferred to the small animal MRI (magnetic resonance imaging) facility for acclimatization. Mice were group-housed in an enriched environment (red transparent plastic boxes, nesting material, paper rolls) until the first behavioral test, after which they were separated due to excessive fighting. Animals were maintained on a 12-hour light/dark cycle with ad libitum access to standard chow and acidified tap water. Room temperature and humidity were monitored and kept at 22 ± 1 °C and 50 - 60%, respectively.

### 2.2 Middle cerebral artery occlusion

Middle cerebral artery occlusion (MCAO) was performed as previously described (53) between 2 and 4 p.m. In summary, anesthesia was induced with 2.5% isoflurane in a 0.7/0.3 N_2_O/O_2_ mix (1 L/min) and maintained at 2% during surgery. Body temperature was kept at ≥36.5 °C using a feedback-controlled heating pad. After a ventral cervical incision, the common carotid artery was ligated below the bifurcation. A silicon-coated filament (Doccol Corporation, size based on body weight) was inserted and advanced to the middle cerebral artery origin. Mice remained awake during occlusion and were reanesthetized for filament withdrawal after 45 min. Wounds were closed with interrupted sutures, and lidocaine gel was applied for pain relief.

Surgery time never exceeded 15 minutes. Animals were kept in a warming chamber overnight with soft chow and recovery gel. Body temperature, weight, and outcome scores (including weight loss, fur condition, behavior, modified Bederson score and breathing, see supplement S1) were assessed daily and animals were euthanized when reaching a sum score of 12 or spinning around their own axis.

To minimize surgical time and anesthesia side effects, cerebral blood flow reduction was not monitored; infarct induction was instead confirmed by MRI after 16 hours.

### 2.3 SorA isolation

SorA, a macrolide natural product, with known anticancer, immune-modulatory, antifungal and antiviral activities, was isolated from the myxobacterium *Sorangium cellulosum*. Purity was confirmed to be greater than 93% using liquid chromatography tandem mass spectrometry (HPLC-MS/MS).

### 2.4 Injections

**Figure 1:**
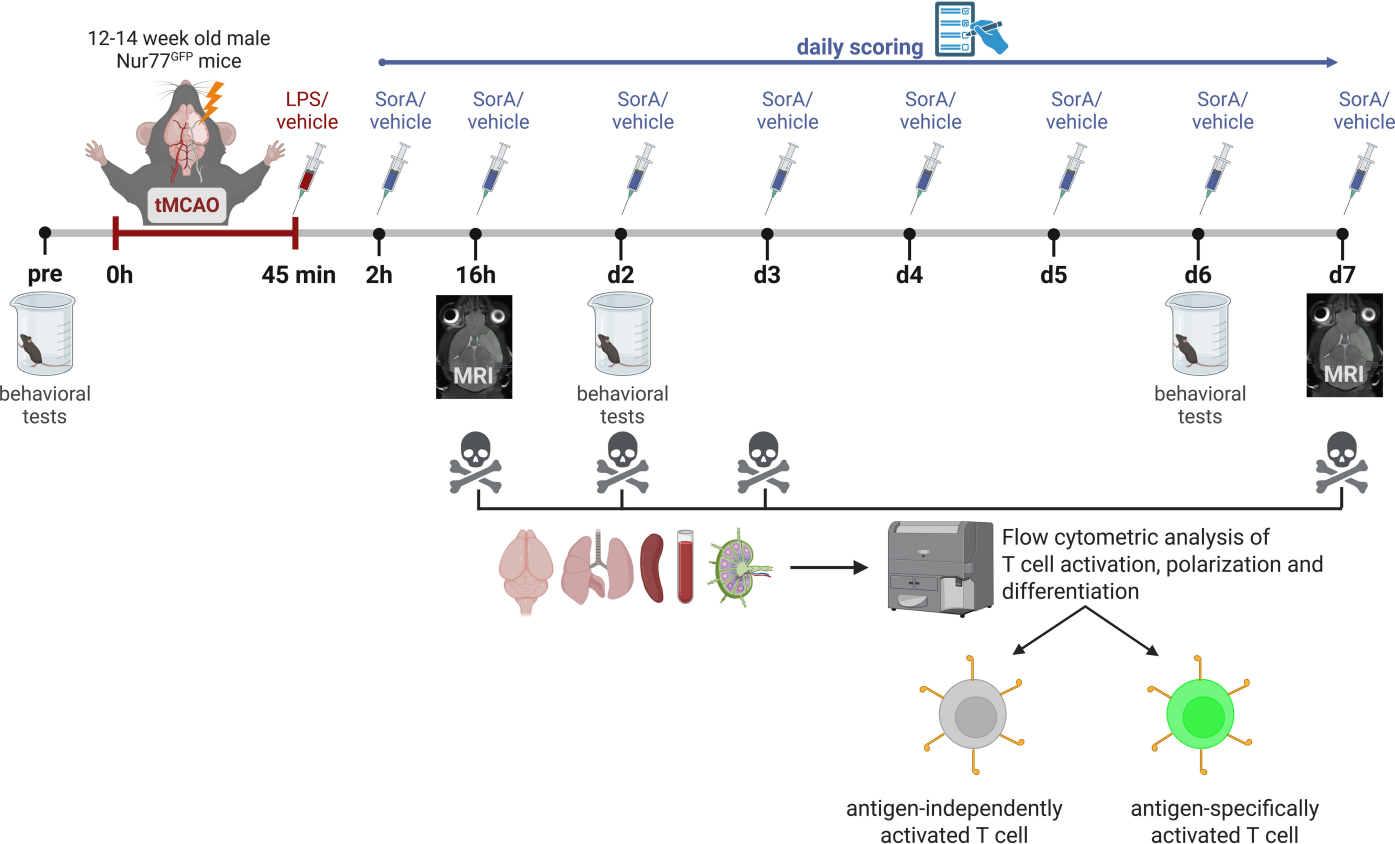
Study design. 12–14 week old male Nur77^GFP^ mice underwent tMCAO, transient middle cerebral artery occlusion. At the timepoint of reperfusion, mice were injected intraperitoneally with LPS (40 µg/kg) or vehicle (PBS). Two hours after tMCAO and daily in the morning, mice were injected intraperitoneally with Soraphen A (SorA, 2.5 µmol/kg) or vehicle (PEG buffer). Magnetic resonance imaging (MRI) was conducted 16 hours and 7 days after tMCAO to verify infarct induction and calculate lesion volumes. Mice were scored daily and a set of behavioral tests (corner tests, cylinder test and inclined plane test) was conducted before as well as 2 and 6 days after tMCAO. At each experimental time point (16 h, 2 d, 3 d, 7 d after MCAO) brain, lungs, spleen, blood and ingiunal lymph nodes (ILN) were harvested and prepared for flow cytometric analysis of T cell activation and polarization, differentiating between antigen-specific and -unspecific T cell activation via GFP expression. To evaluate the impact of SorA treatment on stroke pathology under post-stroke and post-stroke inflammatory conditions associated with infection, we compared treatment groups to assess: the effect of LPS alone (vehicle/vehicle vs. LPS/vehicle), the effect of SorA alone (vehicle/vehicle vs. vehicle/SorA), and the effect of SorA under proinflammatory conditions (LPS/vehicle vs. LPS/SorA) in MCAO mice. Image *created in BioRender. Vogelgesang, A. (2026) https://BioRender.com/pr62lu3*.

At reperfusion, mice were injected intraperitoneally with LPS (40 µg/kg) or vehicle (PBS). Two hours after MCAO and daily in the morning, mice were injected intraperitoneally with SorA (500 µM in PEG buffer [PBS + 5% PEG400 + 2.5% Tween80 + 0.5% EtOH], 5 ml/kg, 2.5 µmol/kg) or vehicle (PEG-buffer). To prevent dehydration, a subcutaneous injection of 0.9% NaCl (10 ml/kg) was administered on the day of the surgery as well as on each following day until day 5 (**Figure 1**).

### 2.5 Magnetic resonance imaging

Brain scans were performed 16 hours post-tMCAO for all mice and at 7 days post-tMCAO for the d7 group using 3D T2-weighted imaging (mouse brain coil, TR = 2000 ms, TE = 37 ms, FoV 19 × 25 mm, thickness 0.45 mm) and diffusion-weighted imaging using a 7T animal MRI (Bruker ClinScan). We selected this time point because it more closely reflects the clinical timeline in stroke patients, while still allowing for reliable assessment of stroke induction and localization by MRI at an early stage.

Anesthetized mice (1-2% isoflurane in 1 L/min oxygen) were kept warm and respiration was monitored during imaging. T2 lesion volumes were assessed by two independent investigators using OsiriX software (version 4.6, Pixmeo, Switzerland), with regions of interest manually selected and volumes calculated semiautomatically. The mean of both investigators’ measurements was used for analysis.

### 2.6 Behavioral testing

To assess the functional outcome, mice underwent 3 different behavioral tests before as well as 2 and 6 days after the MCAO surgery.

In the corner test, mice were placed between two boards arranged in a 30° angle. The number of right and left turns out of 10 turns was counted.

In the cylinder test, mice were placed in a glass cylinder and observed for 3 minutes. The number of wall contacts and the paw used (right only, left only, or both) were recorded.

In the inclined plane test, mice were placed on an adjustable platform. Beginning at 0° (horizontal), the inclination was gradually increased until the animals began to slip, and the angle recorded. The test was performed three times, and the mean angle was calculated.

### 2.7 Cell isolation

At each experimental time point (16 h, 2 d, 3 d, 7 d after MCAO), mice were deeply anesthetized with 3% isoflurane in 1 l/min oxygen and transcardially perfused with HBSS (Hanks’ Balanced Salt Solution; w/o Ca/Mg). Transcardiac perfusion was performed in adaptation of the protocol published by Wu et al. (63). Following a lateral incision below the rib cage, careful dissection separated the liver and gallbladder from the diaphragm. The xiphoid process was grasped using a large hemostat and secured above the mouse’s head to provide traction. A full-length incision was made through the diaphragm, and the rib cage was transected on both sides. Immediately before perfusion, blood was collected from the left ventricle by cardiac puncture (25G needle) and anticoagulated with EDTA. A 24G safety cannula (Introcan) was inserted into the left ventricle, and the needle was withdrawn, leaving only the plastic cannula in place. The vasculature was perfused with cold HBSS via the cannula, and the right atrium was incised for drainage. Perfusion continued until the liver and paws were visibly cleared, typically requiring 20–30 mL of cold HBSS. Following perfusion, the brains, spleens, lungs and inguinal lymph nodes (ILN) were harvested and stored in HBSS (without Ca/Mg) pending cell isolation.

#### 2.7.1 Brain

Following Pösel et al. (64), brains were harvested, hemispheres (ipsi-/contralateral) homogenized and enzymatically digested. Myelin was removed by density centrifugation, and cells were washed with HBSS + 10% FCS before resuspension in HBSS (w/o Ca/Mg) for FACS staining.

#### 2.7.2 Spleen

Spleens were mechanically homogenized through a 70 µm cell strainer (Greiner bio-one), centrifuged and cell pellets resuspended in 2 mL of HBSS (w/o Ca/Mg). Erythrocytes were lysed with 5 mL of ACK (Ammonium-Chloride-Potassium) lysis buffer (Gibco) (10 min, 4 °C). Cells were washed and resuspended in HBSS (w/o Ca/Mg) for FACS staining.

#### 2.7.3 Blood

100 µL of EDTA-anticoagulated blood were used for extracellular FACS staining. Then, erythrocytes were lysed for 10 min at 4 °C in BD FACS lysis solution before intracellular staining. Plasma was prepared from the remaining blood by centrifugation (17 800g, 5 min), frozen at -20 °C and transferred to -80 °C for long-term storage.

#### 2.7.4 Lungs

Lungs were mechanically homogenized through a 100 µm cell strainer (Greiner bio-one). Cell pellets were resuspended in digestion buffer (4 mL HBSS w Ca/Mg + 2.5 µL DNase I + 2 µL Liberase) and incubated at 37 °C for 45 min. After adding 2 mL FCS, the suspension was filtered (70 µm), centrifuged (300 g, 5 min), resuspended in 300 µL FACS buffer, and passed through a 40 µm strainer.

### 2.8 Flow cytometry

Cells were incubated with Zombie Aqua (BioLegend) for dead cell removal. After washing with FACS buffer, samples were incubated with TruStainfcX™ (anti-mouse CD16/32, clone 93, BioLegend) for 10 min at 4 °C to prevent nonspecific staining. For T lymphocyte analysis, cells were subsequently stained with anti-mouse CD45 PerCP-Cy5.5 (clone 30-F11, BioLegend), anti-mouse CD3 AlexaFluor 700 (clone 17A2, BioLegend), anti-mouse CD4 BrilliantViolet 650 (clone RM4-5, BioLegend), anti-mouse CD8a APC/Cy7 (clone 53-6.7, BioLegend), anti-mouse CD25 BrilliantViolet 421 (clone PC61, BioLegend), anti-mouse CD69 PE/Cy5 (clone H1.2F3, BioLegend), and anti-mouse PD-1 (programmed cell death protein 1) BrilliantViolet 605 (clone 29F.1A12, Biolegend) for 30 min at 4 °C in the dark. Cells were washed with FACS buffer and pre-fixed with 1% PFA to preserve the GFP signal. After washing, cells were fixed and permeabilized using the True-Nuclear Transcription Factor Buffer Set (BioLegend) according to the manufacturer’s protocol (fixation time: 30 min). For intracellular staining, cells were incubated with the following antibodies for 30 minutes at room temperature in the dark: anti-human/mouse T-bet PE/Cy7 (clone 4B10, BioLegend), anti-human/mouse GATA-3 AlexaFluor 647 (clone 16E10A23, BioLegend), anti-mouse RORγt PE-CF594 (clone Q31-378, BD), and anti-mouse/rat/human FOXP3 PE (clone 150D, BioLegend). Finally, Trucount beads were added for cell quantification and samples were immediately measured using a BD LSR II flow cytometer (Becton Dickinson, USA). In addition, GFP fluorescence was measured. Analysis of flow cytometry data was performed using FlowJo (version 10.8.1) (for gating strategy see supplement S2).

### 2.9 Cytokine expression

The LEGENDplex Mouse Th Cytokine Panel 12-Plex (BioLegend, REF 741044) was performed according to the manufacturer’s protocol. The standard was performed in duplicates, samples were diluted 1:2 and ran in singlets. Instead of vacuuming, beads were centrifuged down at 1100 rpm for 5 minutes and the supernatant was removed via pipette. Samples were measured at a BD LSR II Flow Cytometer in the YG582 and R670 channels. The number of events to detect was set to 5.000 in total. Data analysis was performed using the LEGENDplex software (version 8) provided by BioLegend.

### 2.10 Sample size calculation

Sample size was calculated for two tailed input in advance with a power of 0.90, alpha of 0.01 and beta of 0.1 *a priori* based on published Soraphen A effect sizes on T cell polarization (40). Secondary endpoints included activation kinetics, immune cell infiltration into peripheral organs, infarct size, and functional outcome. An anticipated dropout rate of 30%, reflecting the severity of the tMCAO model, was incorporated into study planning and was not exceeded during the experiments.

### 2.11 Exclusion criteria and methods to prevent bias

Individual animals were randomly allocated to the treatment groups and observation timepoints by a person not involved in the experiments. Investigators remained blinded to group allocation throughout data acquisition and evaluation.

Every animal had an infarct core, even though the infarct size was sometimes small (mean ± SD: 72.4 ± 24.7 mm^3^). Animals with smaller infarcts were not excluded, reflecting the variability seen in human stroke. To avoid confounding treatment effects with stroke severity, analyses were adjusted for infarct volume.

We included 197 mice in our study. Animals were excluded if the stroke induction was not successful (n=9) or if a stroke was induced in non-middle cerebral artery territory (n=17) based on brain MRI at 16 hours. Additionally, the well-being of animals was assessed based on daily scoring (supplement S1). Only animals that reached their intended experimental endpoint (16 h, 2 d, 3 d, 7 d) were included, while animals that died before the endpoint (n=25) or animals that had to be euthanized before the end of the study due to reaching the humane endpoint score (n=11) were excluded from final data evaluation. These exclusion criteria were decided *a priori*. 6 additional animals were excluded due to urinary infection (n=1), experimental failure (n=1) and lack of GFP expression (n=4). Kaplan-Meier plots are provided in the Supplement (**Supplementary Figure 6**).

For the robustness of the data, we excluded data points from flow cytometric analyses when the parent gate did not include sufficient events for further subgating (<50 events in the parent population). The accordingly available sample size for each time point and analyses is given in the Figure legends. This approach ensures that all data shown and given for interpretation is robust.

### 2.12 Statistical analysis

All statistical analysis was done with R version 4.4.3 and addon packages as “car”, “lmerTest”, “emmeans”, and “ggplot2”. All results are given as mean ± 95% confidence limits if not stated otherwise. The significance level was set to 0.05. Infarct volume was included in all regression models as a covariable to calculate adjusted treatment effects due to potential confounding by infarct volume.

We compared treatment groups to assess the effect of LPS alone (vehicle/vehicle vs. LPS/vehicle), the effect of SorA alone (vehicle/vehicle vs. vehicle/SorA), and the effect of SorA under proinflammatory conditions (LPS/vehicle vs. LPS/SorA) to evaluate SorA’s effects on stroke pathology under post-stroke conditions as well as increased proinflammatory conditions associated with post-stroke infections. We used regression models including the main effects of LPS and SorA and its interaction LPS * SorA. If the interaction was non-significant, p-values are reported for the marginal effects of LPS and SorA, representing independent main effects only. In case of significant interaction, presented p-values reflect the conditional effects of LPS (stratified by SorA treatment) and SorA (stratified by LPS treatment), respectively.

Time was incorporated as an additional factor, and interactions with time were tested. If interactions with time were non-significant (indicating that LPS and SorA effects were constant over time), we report marginal LPS/SorA effects averaged across time points and present the data jointly. Only in case of significant interactions (i.e. effect modifications by timepoint) conditional LPS/SorA effects stratified by time points will be shown.

For outcome parameters with repeated measures over time (body weight, temperature, sum score, behavioral tests) appropriate linear mixed models [LMM] with animal-ID as random effect were used. For outcome parameters with independent animal samples per timepoint (e.g. infarct volume) appropriate linear regression models were applied. For the analysis of flow cytometric data, we employed appropriate (generalized) linear models [(G)LMs]. When GLMs were used, they were specified as Gamma-distribution models with a log-link function. All results are reported on the original parameter scale, with back-transformation applied where necessary.

## 3 Results

All data are available from the authors upon reasonable request.

We report the effects of LPS and SorA treatment on stroke outcomes and T cell responses in the MCAO mouse model. When treatment effects were consistent across time points, data were combined and presented together to improve clarity. All analyses were adjusted for infarct volume to avoid confounding treatment effects with variations in infarct size.

### 3.1 SorA improves functional outcome and mitigates LPS-induced impairments

**Figure 2:**
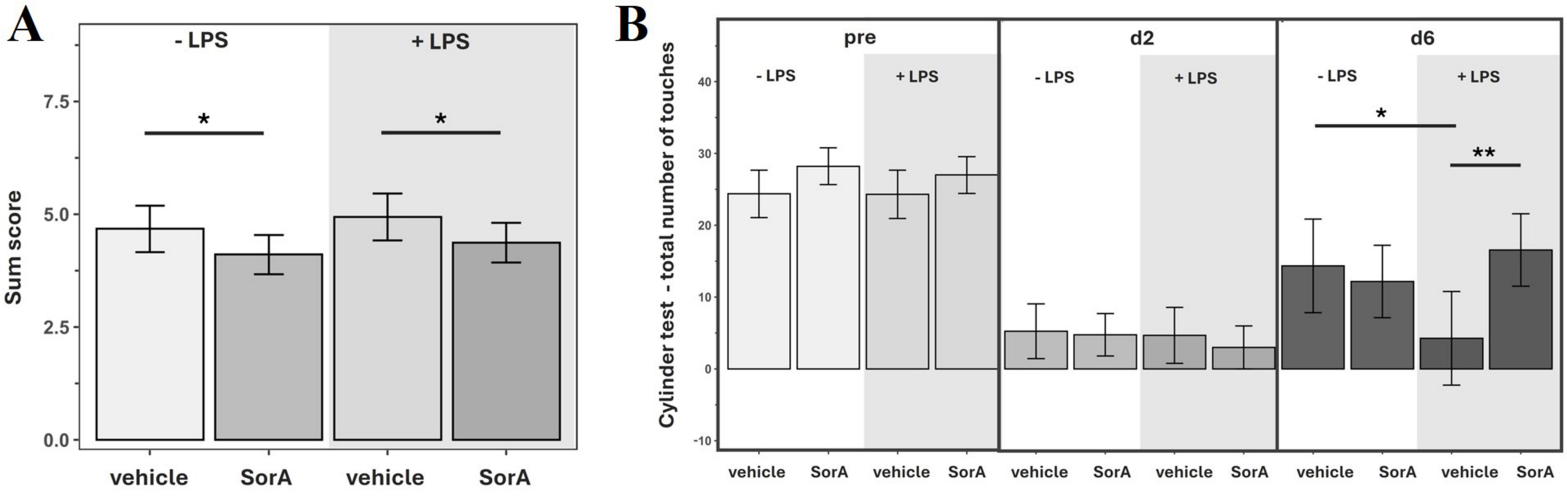
Soraphen A improves functional outcome and compensates LPS-induced effects in mice after transient middle cerebral artery occlusion (tMCAO). Data are adjusted for infarct volume and presented as mean ± 95% confidence limits. Linear mixed models were applied. Models included SorA × LPS interaction (2×2 ANOVA); if non-significant, main effect models including only the independent effects of SorA and LPS were applied. **(A)** Sum score consisting of 5 subscores (weight loss, fur condition, behavior/activity, modified Bederson score, breathing; see supplement S1) of mice after tMCAO. Since no interaction with time was detected, effects were averaged across all measured timepoints. *p=0.0407 (-LPS/vehicle: n=24, -LPS/SorA: n=40, +LPS/vehicle: n=24, +LPS/SorA: n=40). **(B)** Total number of touches in the cylinder test over 3 minutes. *p=0.0320, **p=0.0040 (pre: -LPS/vehicle: n=24, -LPS/SorA: n=40, +LPS/vehicle: n=24, +LPS/SorA: n=40; d2:-LPS/vehicle: n=18, -LPS/SorA: n=30, +LPS/vehicle: n=17, +LPS/SorA: n=29; d7:-LPS/vehicle: n=6, -LPS/SorA: n=10, +LPS/vehicle: n=6, +LPS/SorA: n=10).

LPS-injection of MCAO animals was associated with worse outcome parameters such as lower body weight, a higher proportion of left turns in the corner test as well as fewer touches in the cylinder test 6 days after MCAO compared to vehicle only (**Figure 2**; **Supplementary Figure 7**). LPS did not significantly change the infarct volume (p=0.2043, data not shown).

SorA-injection significantly decreased the sum score of MCAO mice independently of LPS. Compared to animals treated with LPS/veh, mice that received both LPS and SorA showed a higher number of touches in the cylinder test 6 days after MCAO (**Figure 2**). There was no significant effect of SorA on infarct volume (p=0.2363, data not shown).

### 3.2 SorA counteracts LPS-induced peripheral T cell activation

#### 3.2.1 Inguinal lymph nodes

**Figure 3:**
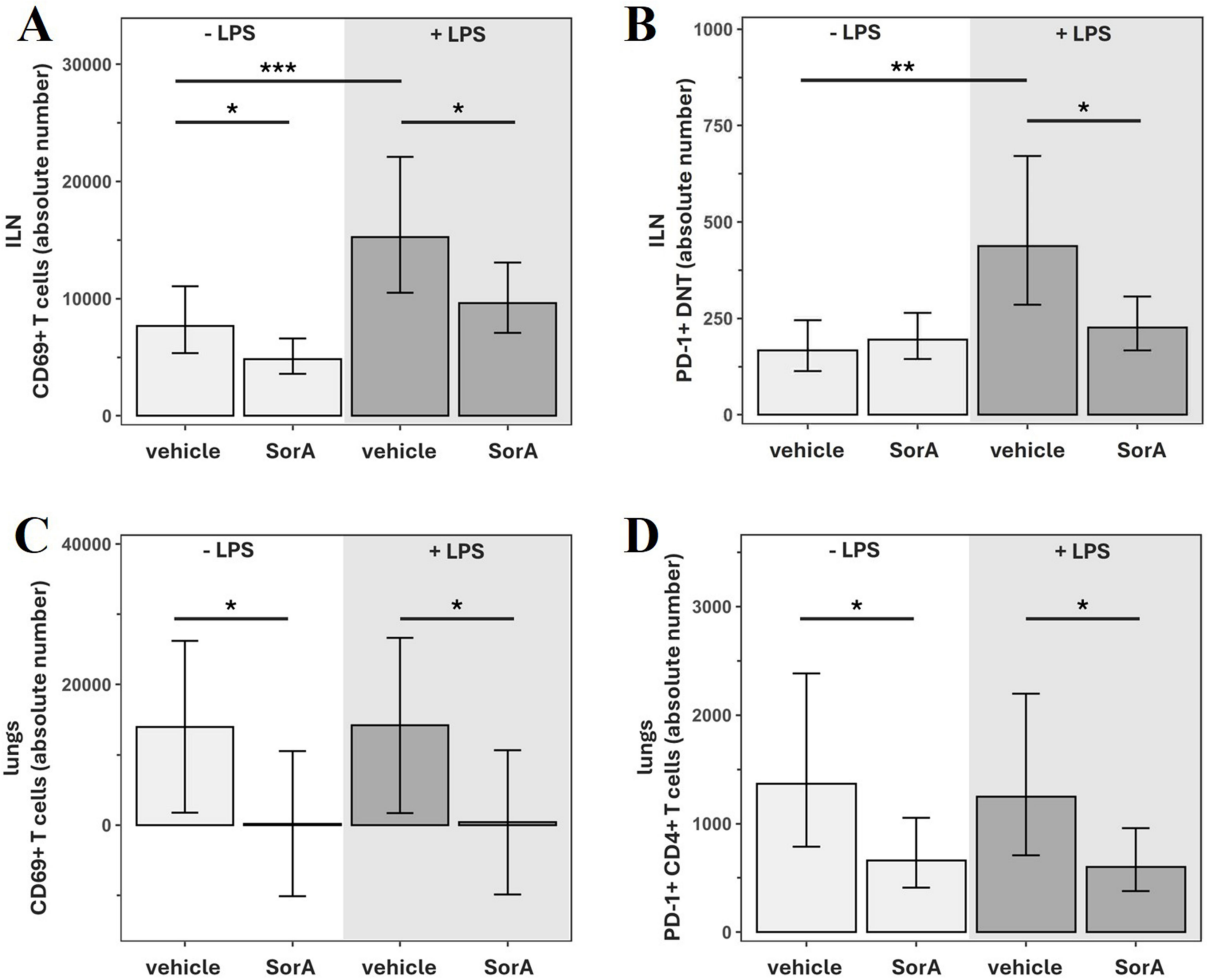
Soraphen A decreases CD69 and PD-1 expression on T cells in ILN, inguinal lymph nodes and lungs and compensates LPS-induced effects in mice after transient middle cerebral artery occlusion (tMCAO). Data are adjusted for infarct volume and presented as mean ± 95% confidence limits. Since no interaction with time was detected, treatment effects are reported as average effects across all measured timepoints. Linear models/generalized linear models (Gamma-distribution models with a log-link function) were applied and results shown on original scale (back-transformed if needed). Models included SorA × LPS interaction (2×2 ANOVA); if non-significant, main effect models including only the independent effects of SorA and LPS were applied. **(A)** Absolute number of CD69^+^ T cells in ILN. ***p=0.0006, *p=0.0235 (-LPS/vehicle: n=24, -LPS/SorA: n=40, +LPS/vehicle: n=24, +LPS/SorA: n=40). **(B)** Absolute number of PD-1^+^ double-negative T cells (CD4^-^/CD8^-^; DNT) in ILN. **p=0.0012, *p=0.0143 (-LPS/vehicle: n=24, -LPS/SorA: n=40, +LPS/vehicle: n=24, +LPS/SorA: n=40). **(C)** Absolute number of CD69^+^ T cells in lungs. *p=0.0424 (-LPS/vehicle: n=24, -LPS/SorA: n=40, +LPS/vehicle: n=24, +LPS/SorA: n=40). **(D)** Absolute number of PD-1^+^ CD4^+^ T cells in lungs. *p=0.0182 (-LPS/vehicle: n=24, -LPS/SorA: n=40, +LPS/vehicle: n=24, +LPS/SorA: n=40).

LPS-injected MCAO mice showed increased activation of peripheral T cells that was most pronounced in the inguinal lymph nodes. There, the absolute numbers of CD25^+^ T cells, CD25^+^CD4^+^ and CD25^+^ DNT were significantly higher than in vehicle-treated MCAO mice (**Supplementary Figures 8A–C**). An increase in the proportion of CD25^+^ T cells within CD4^+^ T cells was also apparent (**Supplementary Figure 8D**). LPS elevated CD69 expression in both absolute numbers and percentage of parent population in overall T cells, CD8^+^ T cells and DNT (**Figure 3A**; **Supplementary Figure 9**). Additionally, the absolute number of PD-1^+^ CD8^+^ T cells and PD-1^+^ DNT in the inguinal lymph nodes was increased in LPS-injected mice (**Figure 3B**; **Supplementary Figure 12B**).

SorA treatment decreased T cell activation in the ILN independently of LPS as reflected by a diminished proportion of CD25^+^ cells within DNT and PD-1^+^ cells within CD4^+^ T cells (**Supplementary Figures 8C, 12A**). Decreased numbers of CD69^+^ T cells, CD69^+^ CD4^+^ and CD69^+^ CD8^+^ as well as a decreased percentage of CD69^+^ cells within the T cell, CD4^+^ and DNT population were seen in SorA-treated mice both in the presence and absence of LPS (**Figure 3A**; **Supplementary Figure 9**). In mice treated with LPS, SorA significantly reduced absolute numbers of CD69^+^ DNTs and PD-1^+^ DNTs, effectively normalizing the LPS-induced increase in T cell activation (**Figure 3B**; **Supplementary Figure 9D**).

#### 3.2.2 Lungs

Since post stroke infections often manifest as pneumonia, we also report changes induced within the lung. In the lungs of LPS-treated MCAO mice, both absolute number and proportion of CD25^+^ DNT were elevated and CD69 expression was increased in CD8^+^ T cells (**Supplementary Figures 8, 10**).

SorA treatment significantly decreased CD69 and PD-1 expression in the lungs regardless of LPS: We observed significantly lower numbers of CD69^+^ T cells, CD69^+^ CD4^+^ and CD69^+^ CD8^+^ T cells as well as fewer PD-1^+^ T cells, PD-1^+^ CD4^+^ and PD-1^+^ CD8^+^ T cells after SorA-injection (**Figure 3**; **Supplementary Figures 10, 12**). The proportion of CD69^+^ cells within the T cell and CD4^+^ T cell compartment was also significantly decreased by SorA injection and the same trend seen for CD8^+^ T cells (p=0.0651) (**Supplementary Figure 10**). In the lungs of SorA-treated mice, PD-1 was expressed by a significantly lower percentage of DNT (p=0.0064) (**Supplementary Figure 12F**).

#### 3.2.3 Spleen

In the spleen, the proportion of CD69^+^ cells within the DNT population was increased in LPS-treated mice (**Supplementary Figure 11A**).

#### 3.2.4 Blood

In the blood of LPS-treated MCAO mice, the proportion of CD69^+^ cells within the DNT population was increased while the absolute number of CD69^+^ DNT was decreased (**Supplementary Figure 11**). Additional SorA treatment increased the absolute number of CD69^+^ DNT and the percentage of CD69^+^ T cells, normalizing the LPS-induced decrease (**Supplementary Figure 11**).

#### 3.2.5 Brain

In contrast to peripheral activation, there were fewer activated T cells in the brain of LPS-treated mice as reflected by decreased CD25^+^ DNT, CD69^+^ CD4^+^ T cells as well as PD1^+^ T cells, PD-1^+^ CD4^+^ and PD-1^+^ CD8^+^ T cells (**Supplementary Figure 13**).

### 3.3 Peripheral GFP expression increased with SorA

**Figure 4:**
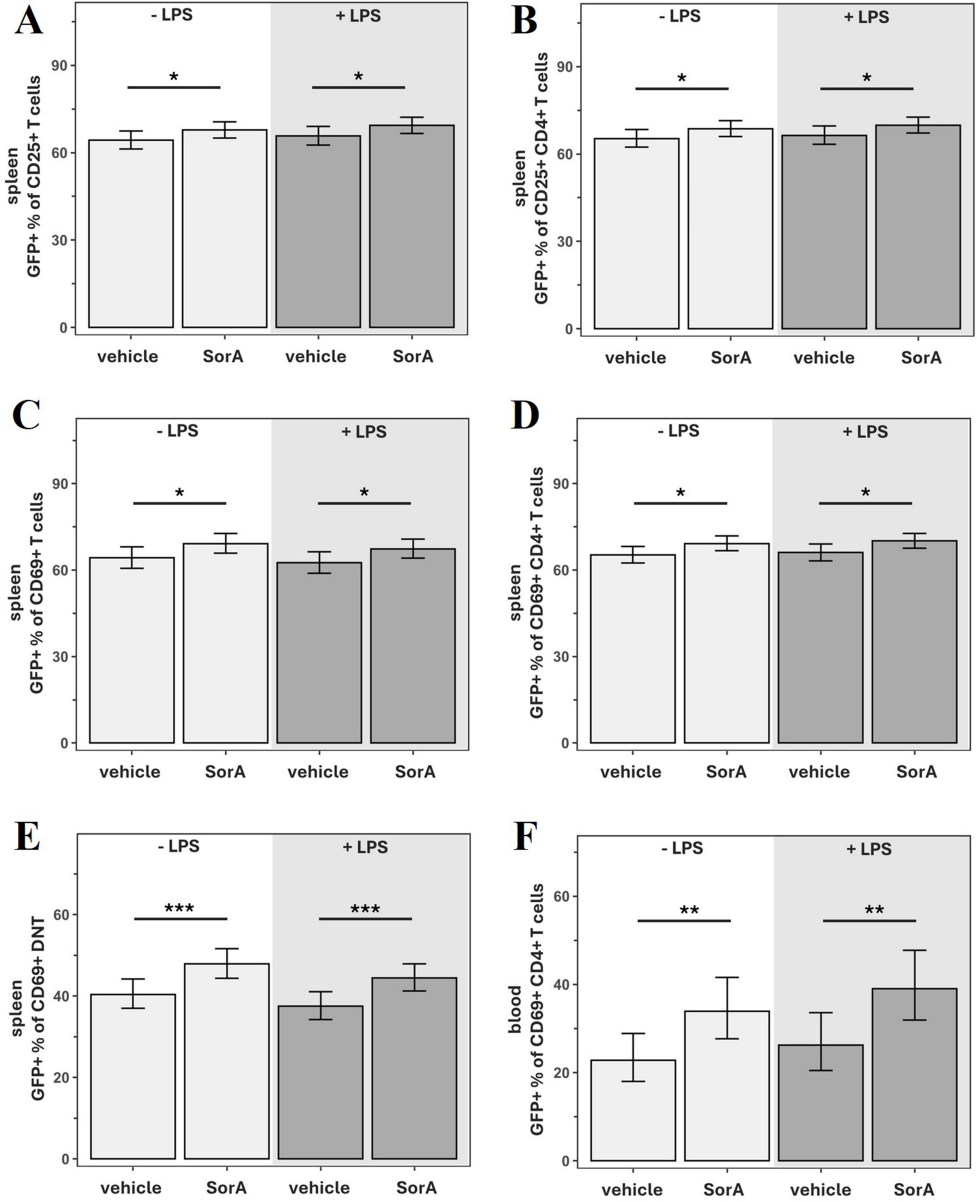
Soraphen A increases GFP expression on T cells in spleen and blood of mice after tMCAO, transient middle cerebral artery occlusion. Data are adjusted for infarct volume and presented as mean ± 95% confidence limits. Since no interaction with time was detected, treatment effects are reported as average effects across all measured timepoints. Linear models/generalized linear models (Gamma-distribution models with a log-link function) were applied and results shown on original scale (back-transformed if needed). Models included SorA × LPS interaction (2×2 ANOVA); if non-significant, main effect models including only the independent effects of SorA and LPS were applied. **(A)** Proportion of antigen-specifically activated (GFP^+^) cells within CD25^+^ T cells in the spleen. * p=0.0457 (-LPS/vehicle: n=24, -LPS/SorA: n=40, +LPS/vehicle: n=24, +LPS/SorA: n=40). **(B)** Proportion of antigen-specifically activated (GFP^+^) cells within CD25^+^ CD4^+^ T cells in the spleen. * p=0.0498 (-LPS/vehicle: n=24, -LPS/SorA: n=40, +LPS/vehicle: n=24, +LPS/SorA: n=40). **(C)** Proportion of antigen-specifically activated (GFP^+^) cells within CD69^+^ T cells in the spleen. * p=0.0232 (-LPS/vehicle: n=24, -LPS/SorA: n=40, +LPS/vehicle: n=24, +LPS/SorA: n=40). **(D)** Proportion of antigen-specifically activated (GFP^+^) cells within CD69^+^ CD4^+^ T cells in the spleen.* p=0.0152 (-LPS/vehicle: n=24, -LPS/SorA: n=40, +LPS/vehicle: n=24, +LPS/SorA: n=40). **(E)** Proportion of antigen-specifically activated (GFP^+^) cells within CD69^+^ double-negative T cells (CD4^-^/CD8^-^; DNT) in the spleen.***p=0.0007 (-LPS/vehicle: n=24, -LPS/SorA: n=40, +LPS/vehicle: n=24, +LPS/SorA: n=40). **(F)** Proportion of antigen-specifically activated (GFP^+^) cells within CD69^+^ CD4^+^ T cells in the blood. **p=0.0030 (-LPS/vehicle: n=23, -LPS/SorA: n=35, +LPS/vehicle: n=21, +LPS/SorA: n=36).

Post-stroke SorA injection significantly increased the percentage of GFP^+^ cells within CD25^+^ T cells and CD25^+^ CD4^+^ T cells as well as within CD69^+^ T cells, CD69^+^ CD4^+^ T cells and CD69^+^ DNT populations in the spleen of MCAO mice independently of LPS (**Figures 4A–E**). The same was true for circulating CD69^+^ CD4^+^ T cells (**Figure 4F**). Due to the low number of recoverable lymphocytes after stroke, analysis of GFP expression was not possible in brain samples.

### 3.4 SorA effects on the Th17/Treg balance

**Figure 5:**
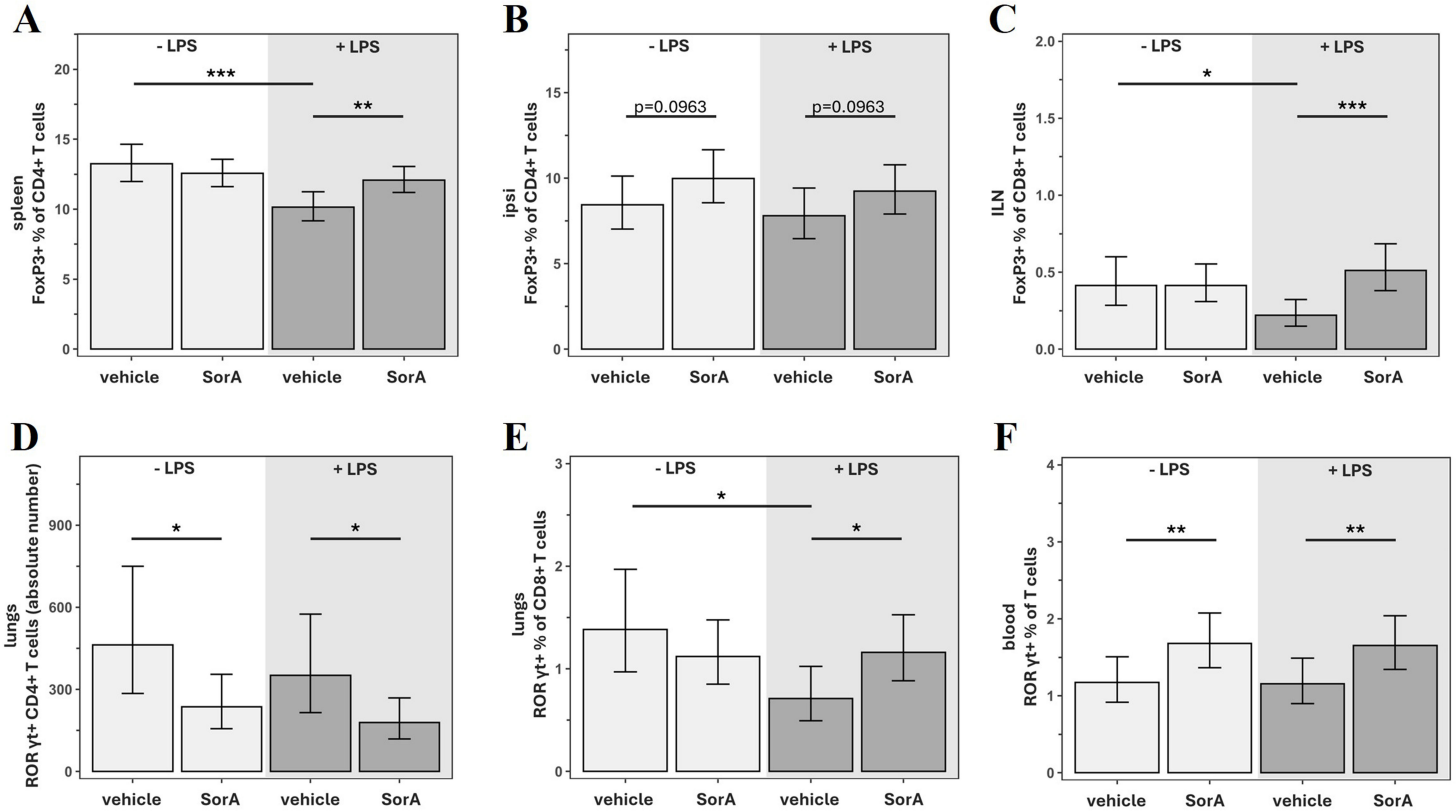
Soraphen A shifts T cell polarization in mice after transient middle cerebral artery occlusion (tMCAO). Data are adjusted for infarct volume and presented as mean ± 95% confidence limits. Since no interaction with time was detected, treatment effects are reported as average effects across all measured timepoints. Linear models/generalized linear models (Gamma-distribution models with a log-link function) were applied and results shown on original scale (back-transformed if needed). Models included SorA × LPS interaction (2×2 ANOVA); if non-significant, main effect models including only the independent effects of SorA and LPS were applied. **(A)** Proportion of FoxP3^+^ cells within CD4^+^ T cells in the spleen. ***p=0.0004, **p= p=0.0083 (-LPS/vehicle: n=24, -LPS/SorA: n=40, +LPS/vehicle: n=24, +LPS/SorA: n=40). **(B)** Proportion of FoxP3^+^ cells within CD4^+^ T cells in the ipsilateral hemisphere. p=0.0963 (-LPS/vehicle: n=24, -LPS/SorA: n=40, +LPS/vehicle: n=23, +LPS/SorA: n=39). **(C)** Proportion of FoxP3^+^ cells within CD8^+^ T cells in the inguinal lymph nodes (ILN). *p=0.0208, ***p=0.0007 (-LPS/vehicle: n=24, -LPS/SorA: n=40, +LPS/vehicle: n=24, +LPS/SorA: n=39). **(D)** Proportion of RORγt^+^ cells within CD4^+^ T cells in the lungs. *p=0.0124 (-LPS/vehicle: n=24, -LPS/SorA: n=40, +LPS/vehicle: n=24, +LPS/SorA: n=40). **(E)** Proportion of RORγt^+^ cells within CD8^+^ T cells in the lungs. *^LPS effect^p=0.0106, *^SorA effect^p=0.0348 (-LPS/vehicle: n=24, -LPS/SorA: n=40, +LPS/vehicle: n=24, +LPS/SorA: n=40). **(F)** Proportion of RORγt^+^ cells within T cells in the blood. **p=0.0096 (-LPS/vehicle: n=24, -LPS/SorA: n=40, +LPS/vehicle: n=24, +LPS/SorA: n=40).

In MCAO mice injected with LPS, animals that also received SorA expressed FoxP3 on a higher proportion of T cells and CD4^+^ T cells in the spleen (**Supplementary Figure 14A**; **Figure 5A**). SorA injection also increased the percentage of FoxP3^+^ CD8^+^ cells in the blood and ILN (**Figure 5C**; **Supplementary Figure 14C**) in LPS-injected mice (**Figure 5C**; **Supplementary Figure 14C**).

Independently of LPS, SorA induced a slight increase in percentage of FoxP3^+^ cells within overall T cells (p=0.0575) and CD4^+^ T cells (p=0.0897) in the lungs (data not shown). Additionally, the percentage of FoxP3^+^ cells within the CD4^+^ T cell population was also slightly increased in the brain while FoxP3^+^ DNT were significantly decreased (**Figure 5B**; **Supplementary Figure 14B**). In the blood of MCAO mice, SorA was associated with higher numbers of RORγt^+^ DNT and an increased proportion of RORγt^+^ cells within T cells regardless of LPS (**Supplementary Figure 14F**; **Figure 5F**). In contrast, the absolute numbers of RORγt^+^ T cells and RORγt^+^ CD4^+^ T cells in the lungs were significantly decreased (**Supplementary Figure 14D**, **Figure 5D**).

In the absence of LPS, SorA decreased RORγt^+^ expression in CD8^+^ T cells in the ILN and the absolute number of RORγt^+^ CD8^+^ T cells in the lungs (**Supplementary Figure 14E**). Under LPS-induced proinflammatory conditions, SorA-injection led to an increase in the percentage of RORγt^+^ CD8^+^ T cells in the lungs (**Figure 5E**).

There was no SorA-dependent change in Treg-cytokine IL-10 or Th17-cytokines IL-17A, IL-17F or IL-22 in the plasma (**Supplementary Figure 15**).

### 3.5 Summary of results

**Figure 6:**
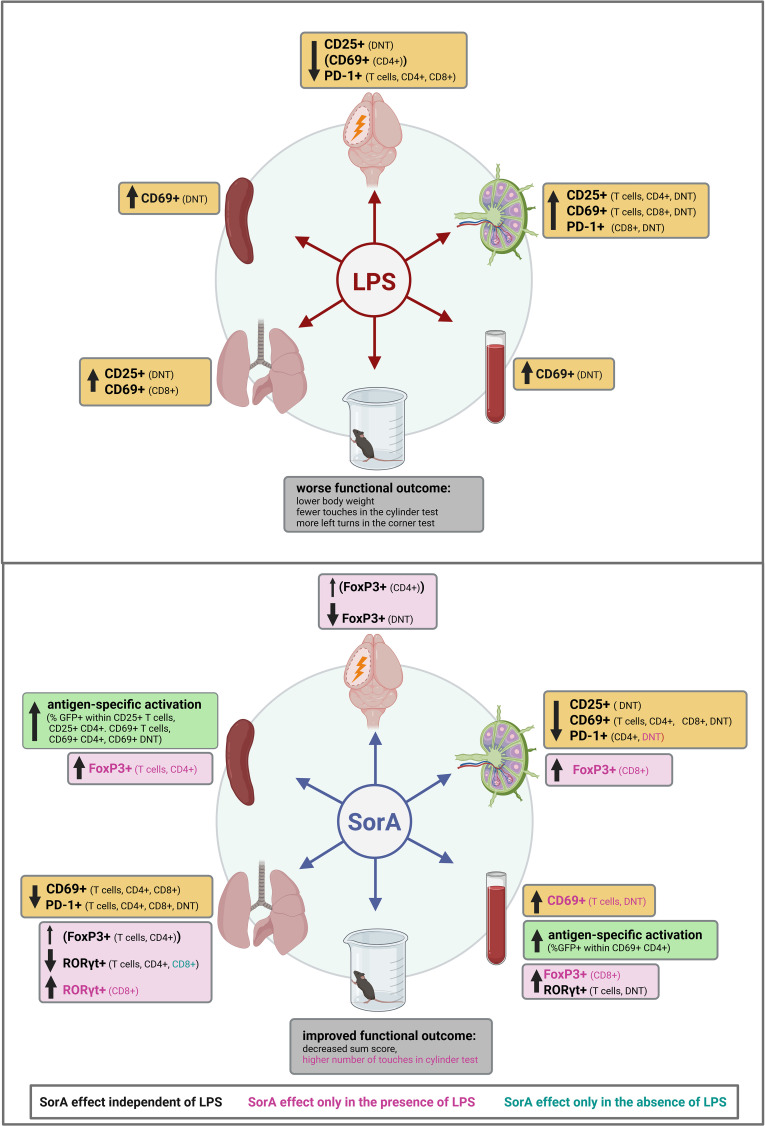
Summary of effects of LPS and SorA, Soraphen A on mice after transient middle cerebral artery occlusion (tMCAO). This graphic summarizes the influence of LPS and SorA on the outcome of mice after tMCAO as well as T cell activation and polarization in brain, lungs, spleen, blood and inguinal lymph nodes (ILN). LPS injection at reperfusion worsened post-MCAO outcomes and increased peripheral T cell activation (CD25, CD69, PD-1) while reducing activation in the brain. SorA improved functional recovery. It reduced T cell activation in ILN and lungs, enhanced antigen-specific activation and elevated FoxP3 expression. RORγt expression was only partially reduced. Image *created in BioRender. Vogelgesang, A. (2026) https://BioRender.com/z5zlsdv*.

LPS injection at reperfusion worsened post-MCAO functional outcomes, as evidenced by greater weight loss, reduced forelimb use in the cylinder test, and increased left-biased turning in the corner test. LPS enhanced peripheral T cell activation, marked by upregulation of CD25, CD69, and PD-1, while concurrently reducing T cell activation within the brain. SorA improved functional outcomes, reflected by a reduced sum score, and reversed LPS-induced inactivity in the cylinder test. In the inguinal lymph nodes and lungs, SorA treatment reduced T cell activation markers (CD25, CD69, PD-1), partially counteracting the LPS-induced increase. Additionally, SorA enhanced antigen-specific T cell activation, particularly in the spleen. FoxP3 expression in both CD4^+^ and CD8^+^ T cell subsets was elevated by SorA, most notably in LPS-treated mice, while RORγt expression showed only a partial reduction (e.g. in the lungs) (**Figure 6**).

## 4 Discussion

SorA, an immunomodulatory substance that promotes Treg polarization while decreasing Th17, has shown promising results in the EAE model of multiple sclerosis and the MCAO model of ischemic stroke (40, 46). Expanding on previous research, we examine the impact of SorA on post-stroke T cell activation and polarization in both peripheral compartments and the brain. Utilizing the Nur77^GFP^ mouse model, we differentiate between antigen-specific T cell activation and unspecific activation driven by proinflammatory stimuli within a natural receptor repertoire.

### 4.1 LPS injection increases peripheral T cell activation and worsens functional outcome

To evaluate SorA’s potential benefits under increased proinflammation associated with post-stroke infection, we employed intraperitoneal LPS injection, a well-established endotoxemia model known to induce systemic inflammation, neuroinflammation, and cognitive impairment (47–50). LPS levels are elevated in the blood of stroke patients and MCAO mice, correlating with poor outcomes and high mortality (65–70). Consistently, our findings show that LPS worsens functional outcomes and increases peripheral T cell activation, marked by high CD69 expression (**Figures 2B**, **3**; **Supplementary Figures 7–12**). This effect was most pronounced in the ILN (likely due to their proximity to the injection site) but also conceivable in other compartments. An intriguing finding of our study is the apparent divergence between peripheral and central T cell activation following LPS administration. While LPS markedly increased activation markers such as CD69 and PD-1 in peripheral compartments, T cell activation within the brain was reduced (**Supplementary Figure 13**). This observation highlights a compartment-specific immune response and may reflect several non-mutually exclusive mechanisms. First, ischemic stroke induces a well-described state of systemic immunosuppression, which may differentially affect immune cell function in the CNS versus the periphery. Second, despite systemic activation, the recruitment of activated T cells into the brain may be limited by altered trafficking dynamics, including changes in chemokine gradients or adhesion molecule expression. Finally, local immunoregulatory mechanisms within the CNS, including microglial activation states and anti-inflammatory cytokine signaling, may actively suppress T cell activation within the brain parenchyma. Together, these findings suggest that systemic inflammation does not directly translate into increased neuroinflammation at each time point, but rather results in a spatially compartmentalized immune response. This dissociation may represent an adaptive mechanism to limit secondary tissue damage in the vulnerable post-ischemic brain. Noteworthily, we used a single low-dose LPS injection (40 µg/kg) during reperfusion, whereas studies reporting neuroinflammation employed repeated injections at significantly higher doses (250 µg/kg – 1 mg/kg) (50).

### 4.2 Soraphen A slightly increases FoxP3 expression and improves outcome parameters

Consistent with previous research findings (40, 46) we report an increase of regulatory FoxP3^+^ T cells, especially in the CD4^+^ population, in the spleen of SorA-treated mice (**Figure 5**). This effect was only seen in mice that also received LPS injections, suggesting SorA may be more potent under proinflammatory conditions and possibly reverse LPS-effects. Consistently, the beneficial effects of SorA on outcome parameters were more pronounced with additional LPS injection: while SorA reduced the sum score in MCAO mice regardless of LPS, only the SorA + LPS group showed improved activity in the cylinder test compared to LPS alone.

Although SorA treatment did not significantly reduce infarct volume (p = 0.2363), it led to notable improvements in neurological function. The functional benefit may reflect improved tissue quality and network function rather than reduced lesion size. T2-weighted MRI-derived infarct volumes are strongly influenced by tissue water content and edema and therefore represent an indirect estimate of structural injury rather than a direct measure of cell death (71). Generally, infarct volume is an important structural readout but an imperfect surrogate of neurological recovery, as functional outcome also depends on lesion location, white matter integrity, network connectivity, and the capacity for peri-infarct plasticity (72). One possible explanation is that SorA improved the functional state of peri-infarct tissue without measurably altering lesion size, for example by modulating neuroinflammation and thereby influencing glial reactivity, structural plasticity, and remyelination-related repair processes (73, 74). Additionally, SorA exerts systemic and immunomodulatory effects that may lead to reduction of systemic inflammation (40, 75). Regulatory T cells, modulated by SorA, suppress excessive neuroinflammation, limit secondary tissue damage in the penumbra and foster a pro-regenerative environment through cytokines like IL-10 and TGF-β, supporting synaptic remodeling, axonal sprouting, and network reorganization (33, 35, 37, 40, 55, 56, 76). Together, these mechanisms can enhance functional recovery independently of infarct volume.

While the study was prospectively powered for the primary immunological endpoint, secondary chronic functional analyses may be underpowered and should therefore be interpreted cautiously. Moreover, because animals with the most severe acute injury are more likely to die or reach humane endpoints early, survivorship bias cannot be fully excluded. In our study, however, exclusions occurred predominantly within the first 24 h after MCAO and were not differentially distributed across treatment groups, as supported by Kaplan-Meier and competing risk analyses showing no group-specific differences in survival or exclusion rates (**Supplementary Figure 6**). In addition, infarct volumes did not differ between treatment groups, arguing against a systematic imbalance in stroke severity among animals included in the final analyses. Together, these findings suggest that the principal immunological conclusions are unlikely to be solely driven by selective survival, although this limitation should be considered when interpreting chronic outcome measures. SorA injection did not significantly increase CD4^+^ Tregs in the brain, although an upward trend was visible (**Figure 5B**). Post-stroke, Tregs accumulate in the ipsilateral hemisphere particularly in the chronic phase (14–30 days after MCAO) (77), contributing to neurological recovery and white matter repair (37, 56). Since we assessed SorA’s effects only within the first 7 days after MCAO, long-term studies are warranted.

In addition to CD4^+^ Treg, we found a higher proportion of FoxP3^+^ CD8^+^ T cells in the blood and ILN (**Figure 5C**; **Supplementary Figure 14C**). Analogously to their CD4^+^ counterparts, FoxP3^+^ CD8^+^ represent a regulatory subpopulation characterized by immunosuppressive features such as production of anti-inflammatory cytokines and high expression of inhibitory cell surface molecules (78). Independently of LPS-injection we observed a decline in the proportion of FoxP3^+^ cells within brain DNTs after SorA application (**Supplementary Figure 14B**). As it remains unclear whether FoxP3^+^ DNTs possess suppressive functions similar to their CD4^+^ and CD8^+^ counterparts, it remains speculative whether this exacerbates or dampens the local inflammation. However, a recent study highlighted FoxP3^+^ DNT as a potential therapeutic target in HIV (79), underscoring the need for further investigation into the role of this subset.

A SorA-induced reduction of RORγt-expressing T cells, including RORγt^+^ Th17 cells, was observed only in the lungs (**Figures 5D, E**; **Supplementary Figures 14D, E**). Additionally, the percentage of RORγt-expressing CD8^+^ T cells was increased in the lungs of SorA-treated mice under LPS-induced proinflammation. This cell subset has been suggested to show an exhausted phenotype, as they are terminally differentiated and have impaired cytotoxic functions (80). While suppression of Th17 responses may help alleviate CNS inflammation and reduce tissue damage, it could also compromise pulmonary antimicrobial defenses. Th17 cells in the blood and brain have been identified as detrimental after stroke (20, 22, 25, 29, 31, 81). However, their role in the lungs might be more complex, as they play a critical role in mucosal immunity and defense against extracellular bacteria involved in post-stroke infections such as pneumonia (82–87). Indeed, SorA treatment has led to an increased susceptibility and dissemination of the gut-infecting *Citrobacter rodentium*, while reducing Th1/Th17-mediated tissue damage (83). Our findings underscore the need to consider peripheral effects of immunomodulatory treatments, which may lead to serious complications. As LPS-injection mimics only the inflammatory response associated with infection and does not capture bacterial replication or full pathogen-specific immunity, validating safety in live bacterial pneumonia or infection models will be essential before considering translation to human stroke patients. Incorporating common stroke comorbidities, such as atherosclerosis, hypertension, or diabetes, would further enhance the translational relevance of these findings. For potential clinical application, SorA treatment may also require prophylactic antibiotic strategies to mitigate the risk of infection.

In contrast to previous studies (40, 46), we found an increased proportion of RORγt^+^ cells among circulating T cells (**Figure 5F**), with no significant change in RORγt expression on CD4^+^ T cells. The observed decrease of RORγt^+^ Th17 cells in lung tissue alongside a relative increase in peripheral blood could potentially be explained by several, not mutually exclusive, mechanisms. Differences in tissue microenvironments – such as oxygen tension, nutrient availability, and local metabolic conditions – may influence T cell differentiation, stability, or survival in a compartment-specific manner. Similarly, variations in the local cytokine milieu might differentially regulate the maintenance of Th17 cells across tissues. In addition, it is possible that changes in chemokine or adhesion molecule expression alter Th17 cell trafficking, for example by affecting tissue retention, egress, or recruitment. Such mechanisms could contribute to an apparent reduction in lung tissue and a relative accumulation in the circulation. Finally, given the well-described phenotypic plasticity of Th17 cells, it cannot be excluded that RORγt expression is downregulated within the tissue or that cells undergo functional reprogramming, which would not necessarily be reflected by changes in circulating populations. At present, these interpretations remain speculative and require direct experimental validation.

Additionally, we observed fewer SorA-mediated changes in Tregs and behavioral outcome (**Figure 2**) compared to previous studies (40, 46), and no detectable changes in plasma levels of Treg- or Th17-associated cytokines (**Supplementary Figure 15**). Similarly, Mamareli et al. reported no significant differences in Th17 or Treg frequencies between SorA-treated and control groups (83). Differences in MCAO induction, treatment protocols (including different concentrations of SorA and different vehicles), time points of sample collection and varying assays and outcome measures may partly explain the discrepancies. Moreover, uncontrolled environmental factors at different research facilities could contribute. For instance, differences in the mice’s microbiome can influence levels of bacterial metabolites such as short-chain fatty acids, which are known to modulate T cell function and reduce neuroinflammation (88–90).

### 4.3 SorA treatment decreased peripheral T cell activation while promoting antigen-specific activation

Beyond its effects on T cell polarization, SorA treatment significantly reduced peripheral T cell activation, measured by the expression of activation markers CD25, CD69 and PD-1. CD69 and PD-1 were downregulated in the lungs and ILN, and SorA partially reversed LPS-induced T cell activation (**Figure 3**, **Supplementary Figures 9–12**). This suggests SorA mitigates excessive inflammation and may reduce inflammation-driven tissue damage, aligning with the improved outcomes observed in SorA-treated mice. However, peripheral immunosuppression could also heighten the risk of post-stroke infections, as discussed before (4.2.).

SorA increased antigen-specific T cell activation in the spleen of MCAO mice while there was no difference in overall activation marker expression (**Figure 4**). An increase in antigen-specific T cell activation likely promotes clonal expansion and may counteract the post-stroke reduction in splenocyte numbers, which is typically associated with poorer outcomes (91). However, further research is needed to confirm this relationship. Depending on what their cognate antigen is, antigen-specifically activated T cells might strengthen the peripheral immune response or infiltrate the brain. A limitation of this study is that antigen-specific T cell infiltration and activation could not be directly assessed in brain tissue due to the low number of recoverable lymphocytes after stroke. Moreover, the use of multiparametric intracellular flow cytometry required for detailed characterization of T cell polarization limits direct translation to tissue-based imaging approaches with comparable phenotypic resolution. Although Soraphen A increased antigen-specifically activated T cells in the periphery, their infiltration into the brain and contribution to the local immune response remain unclear. Future studies should therefore assess intracerebral antigen-specific T cell responses, define their antigenic targets, and characterize their polarization and functional states *in situ*. High-dimensional tissue imaging and complementary approaches such as cytokine profiling or transcriptomic analysis will be important to clarify the spatial distribution and mechanistic relevance of these cells within the ischemic microenvironment analysehemic.

### 4.4 Summary

This study reveals that SorA modulates systemic and organ-specific T cell responses in experimental ischemic stroke. We demonstrate, that SorA improves post-stroke functional outcomes particularly under LPS-induced proinflammatory conditions, suggesting potential protective effects in post-stroke infection. Since the LPS model only resembles the increased proinflammation associated with post-stroke infections, aspects like pathogen clearance will need to be addressed in infection models before clinical translation. Our findings show that SorA increases regulatory T cells in LPS-treated mice and counteracts LPS-induced T cell activation and behavioral deficits. Furthermore, we identify a shift toward antigen-specific, T cell receptor-mediated activation in the peripheral compartments of SorA-treated mice, highlighting its potential role in fine-tuning post-stroke immune responses and extending the therapeutic window into the subacute phase of stroke.

Our study significantly expands upon a previous report demonstrating SorA’s benefits in experimental stroke by offering critical mechanistic insights and addressing comorbid conditions such as post-stroke infections. Additionally, it provides robust validation of SorA’s therapeutic potential through independent replication at a second experimental site.

## Data Availability

The raw data supporting the conclusions of this article will be made available by the authors, without undue reservation.

## References

[1] Gelderblom M Leypoldt F Steinbach K Behrens D Choe CU Siler DA . Temporal and spatial dynamics of cerebral immune cell accumulation in stroke. Stroke. (2009) 40:1849–57. doi: 10.1161/strokeaha.108.534503. PMID: 19265055

[2] Giraud M Cho TH Nighoghossian N Maucort-Boulch D Deiana G Østergaard L . Early blood brain barrier changes in acute ischemic stroke: a sequential MRI study. J Neuroimaging. (2015) 25:959–63. doi: 10.1111/jon.12225. PMID: 25702824

[3] Hoffmann A Dege T Kunze R Ernst AS Lorenz H Böhler LI . Early blood-brain barrier disruption in ischemic stroke initiates multifocally around capillaries/venules. Stroke. (2018) 49:1479–87. doi: 10.1161/strokeaha.118.020927. PMID: 29760276

[4] Kleinschnitz C Schwab N Kraft P Hagedorn I Dreykluft A Schwarz T . Early detrimental T-cell effects in experimental cerebral ischemia are neither related to adaptive immunity nor thrombus formation. Blood. (2010) 115:3835–42. doi: 10.1182/blood-2009-10-249078. PMID: 20215643

[5] Schuhmann MK Langhauser F Kraft P Kleinschnitz C . B cells do not have a major pathophysiologic role in acute ischemic stroke in mice. J Neuroinflamm. (2017) 14:112. doi: 10.1186/s12974-017-0890-x. PMID: 28576128 PMC5457733

[6] Yilmaz G Arumugam TV Stokes KY Granger DN . Role of T lymphocytes and interferon-gamma in ischemic stroke. Circulation. (2006) 113:2105–12. doi: 10.1161/circulationaha.105.593046. PMID: 16636173

[7] Liesz A Zhou W Mracskó É Karcher S Bauer H Schwarting S . Inhibition of lymphocyte trafficking shields the brain against deleterious neuroinflammation after stroke. Brain. (2011) 134:704–20. doi: 10.1093/brain/awr008. PMID: 21354973

[8] Shichita T Sugiyama Y Ooboshi H Sugimori H Nakagawa R Takada I . Pivotal role of cerebral interleukin-17-producing gammadeltaT cells in the delayed phase of ischemic brain injury. Nat Med. (2009) 15:946–50. doi: 10.1038/nm.1999. PMID: 19648929

[9] Dirnagl U Klehmet J Braun JS Harms H Meisel C Ziemssen T . Stroke-induced immunodepression. Stroke. (2007) 38:770–3. doi: 10.1161/01.str.0000251441.89665.bc. PMID: 17261736

[10] Haeusler KG Schmidt WUH Föhring F Meisel C Helms T Jungehulsing GJ . Cellular immunodepression preceding infectious complications after acute ischemic stroke in humans. Cerebrovasc Dis. (2008) 25:50–8. doi: 10.1159/000111499. PMID: 18033958

[11] Liu Q Jin WN Liu Y Shi K Sun H Zhang F . Brain ischemia suppresses immunity in the periphery and brain via different neurogenic innervations. Immunity. (2017) 46:474–87. doi: 10.1016/j.immuni.2017.02.015. PMID: 28314594

[12] Meisel C Schwab JM Prass K Meisel A Dirnagl U . Central nervous system injury-induced immune deficiency syndrome. Nat Rev Neurosci. (2005) 6:775–86. doi: 10.1038/nrn1765. PMID: 16163382

[13] Prass K Meisel C Höflich C Braun J Halle E Wolf T . Stroke-induced immunodeficiency promotes spontaneous bacterial infections and is mediated by sympathetic activation reversal by poststroke T helper cell type 1–like immunostimulation. J Exp Med. (2003) 198:725–36. doi: 10.1084/jem.20021098. PMID: 12939340 PMC2194193

[14] Vogelgesang A May VEL Grunwald U Bakkeboe M Langner S Wallaschofski H . Functional status of peripheral blood T-cells in ischemic stroke patients. PloS One. (2010) 5:e8718. doi: 10.1371/journal.pone.0008718. PMID: 20090932 PMC2806837

[15] Aslanyan S Weir CJ Diener HC Kaste M Lees KR GAIN International Steering Committee and Investigators . Pneumonia and urinary tract infection after acute ischaemic stroke: a tertiary analysis of the GAIN International trial. Eur J Neurol. (2004) 11:49–53. doi: 10.1046/j.1468-1331.2003.00749.x. PMID: 14692888

[16] Kwan J Hand P . Infection after acute stroke is associated with poor short-term outcome. Acta Neurol Scand. (2007) 115:331–8. doi: 10.1111/j.1600-0404.2006.00783.x. PMID: 17489944

[17] Westendorp WF Nederkoorn PJ Vermeij JD Dijkgraaf MG de Beek D . Post-stroke infection: a systematic review and meta-analysis. BMC Neurol. (2011) 11:110. doi: 10.1186/1471-2377-11-110. PMID: 21933425 PMC3185266

[18] Sacks D Baxter B Campbell BCV Carpenter JS Cognard C Dippel D . Multisociety consensus quality improvement revised consensus statement for endovascular therapy of acute ischemic stroke: from the American Association of Neurological Surgeons (AANS), American Society of Neuroradiology (ASNR), Cardiovascular and Interventional Radiology Society of Europe (CIRSE), Canadian Interventional Radiology Association (CIRA), Congress of Neurological Surgeons (CNS), European Society of Minimally Invasive Neurological Therapy (ESMINT), European Society of Neuroradiology (ESNR), European Stroke Organization (ESO), Society for Cardiovascular Angiography and Interventions (SCAI), Society of Interventional Radiology (SIR), Society of NeuroInterventional Surgery (SNIS), and World Stroke Organization (WSO). J Vasc Interv Radiol. (2018) 29:441–53. doi: 10.1016/j.jvir.2013.05.050. PMID: 29478797

[19] Powers WJ Rabinstein AA Ackerson T Adeoye OM Bambakidis NC Becker K . Guidelines for the early management of patients with acute ischemic stroke: 2019 update to the 2018 guidelines for the early management of acute ischemic stroke: a guideline for healthcare professionals from the American Heart Association/American Stroke Association. Stroke. (2019) 50:e344–418. doi: 10.1161/str.0000000000000211. PMID: 31662037

[20] Dolati S Ahmadi M Khalili M Taheraghdam AA Siahmansouri H Babaloo Z . Peripheral Th17/Treg imbalance in elderly patients with ischemic stroke. Neurol Sci. (2018) 39:647–54. doi: 10.1007/s10072-018-3250-4. PMID: 29353353

[21] Li Q Wang Y Yu F Wang YM Zhang C Hu C . Peripheral Th17/Treg imbalance in patients with atherosclerotic cerebral infarction. Int J Clin Exp Pathol. (2013) 6:1015–27. PMC3657353 23696918

[22] Liu X Kenkare K Li S Desai V Wong J Luo X . Increased Th17/Treg ratio in poststroke fatigue. Mediators Inflammation. (2015) 2015:931398. doi: 10.1155/2015/931398. PMID: 26166952 PMC4488542

[23] Huppert J Closhen D Croxford A White R Kulig P Pietrowski E . Cellular mechanisms of IL-17-induced blood-brain barrier disruption. FASEB J. (2010) 24:1023–34. doi: 10.1096/fj.09-141978. PMID: 19940258

[24] Kebir H Kreymborg K Ifergan I Dodelet-Devillers A Cayrol R Bernard M . Human TH17 lymphocytes promote blood-brain barrier disruption and central nervous system inflammation. Nat Med. (2007) 13:1173–5. doi: 10.1038/nm1651. PMID: 17828272 PMC5114125

[25] Lu T Ma L Xu Q Wang X . Blood Th17 cells and IL-17A as candidate biomarkers estimating the progression of cognitive impairment in stroke patients. J Clin Lab Anal. (2022) 36:e24581. doi: 10.1002/jcla.24581. PMID: 35808926 PMC9396181

[26] Ni P Dong H Wang Y Zhou Q Xu M Qian Y . IL-17A contributes to perioperative neurocognitive disorders through blood-brain barrier disruption in aged mice. J Neuroinflamm. (2018) 15:332. doi: 10.1186/s12974-018-1374-3. PMID: 30501622 PMC6267879

[27] Ribeiro M Brigas HC Temido-Ferreira M Pousinha PA Regen T Santa C . Meningeal γδ T cell-derived IL-17 controls synaptic plasticity and short-term memory. Sci Immunol. (2019) 4:eaay5199. doi: 10.1126/sciimmunol.aay5199. PMID: 31604844 PMC6894940

[28] Wang D Zhao Y Wang G Sun B Kong Q Zhao K . IL-17 potentiates neuronal injury induced by oxygen-glucose deprivation and affects neuronal IL-17 receptor expression. J Neuroimmunol. (2009) 212:17–25. doi: 10.1016/j.jneuroim.2009.04.007. PMID: 19457561

[29] Yu S Cui W Han J Chen J Tao W . Longitudinal change of Th1, Th2, and Th17 cells and their relationship between cognitive impairment, stroke recurrence, and mortality among acute ischemic stroke patients. J Clin Lab Anal. (2022) 36:e24542. doi: 10.1002/jcla.24542. PMID: 35689536 PMC9280005

[30] Li GZ Zhong D Yang LM Sun B Zhong ZH Yin YH . Expression of interleukin-17 in ischemic brain tissue. Scand J Immunol. (2005) 62:481–6. doi: 10.1111/j.1365-3083.2005.01683.x. PMID: 16305645

[31] Luo Y Zhou Y Xiao W Liang Z Dai J Weng X . Interleukin-33 ameliorates ischemic brain injury in experimental stroke through promoting Th2 response and suppressing Th17 response. Brain Res. (2015) 1597:86–94. doi: 10.1016/j.brainres.2014.12.005. PMID: 25500143

[32] Kleinschnitz C Kraft P Dreykluft A Hagedorn I Göbel K Schuhmann MK . Regulatory T cells are strong promoters of acute ischemic stroke in mice by inducing dysfunction of the cerebral microvasculature. Blood. (2013) 121:679–91. doi: 10.1182/blood-2012-04-426734. PMID: 23160472 PMC3790947

[33] Liesz A Suri-Payer E Veltkamp C Doerr H Sommer C Rivest S . Regulatory T cells are key cerebroprotective immunomodulators in acute experimental stroke. Nat Med. (2009) 15:192–9. doi: 10.1038/nm.1927. PMID: 19169263

[34] Brea D Agulla J Rodríguez-Yáñez M Barral D Ramos-Cabrer P Campos F . Regulatory T cells modulate inflammation and reduce infarct volume in experimental brain ischaemia. J Cell Mol Med. (2014) 18:1571–9. doi: 10.1111/jcmm.12304. PMID: 24889329 PMC4190903

[35] Liesz A Zhou W Na SY Hämmerling GJ Garbi N Karcher S . Boosting regulatory T cells limits neuroinflammation in permanent cortical stroke. J Neurosci. (2013) 33:17350–62. doi: 10.1523/jneurosci.4901-12.2013. PMID: 24174668 PMC6618366

[36] Na SY Mracsko E Liesz A Hünig T Veltkamp R . Amplification of regulatory T cells using a CD28 superagonist reduces brain damage after ischemic stroke in mice. Stroke. (2015) 46:212–20. doi: 10.1161/strokeaha.114.007756. PMID: 25378432

[37] Shi L Sun Z Su W Xu F Xie D Zhang Q . Treg cell-derived osteopontin promotes microglia-mediated white matter repair after ischemic stroke. Immunity. (2021) 54:1527–1542.e8. doi: 10.1016/j.immuni.2021.04.022. PMID: 34015256 PMC8282725

[38] Schuhmann MK Kraft P Stoll G Lorenz K Meuth SG Wiendl H . CD28 superagonist-mediated boost of regulatory T cells increases thrombo-inflammation and ischemic neurodegeneration during the acute phase of experimental stroke. J Cereb Blood Flow Metab. (2015) 35:6–10. doi: 10.1038/jcbfm.2014.175. PMID: 25315859 PMC4294400

[39] Barbi J Pardoll D Pan F . Metabolic control of the Treg/Th17 axis. Immunol Rev. (2013) 252:52–77. doi: 10.1111/imr.12029. PMID: 23405895 PMC3576873

[40] Berod L Friedrich C Nandan A Freitag J Hagemann S Harmrolfs K . De novo fatty acid synthesis controls the fate between regulatory T and T helper 17 cells. Nat Med. (2014) 20:1327–33. doi: 10.1038/nm.3704 25282359

[41] Lee J Walsh MC Hoehn KL James DE Wherry EJ Choi Y . Regulator of fatty acid metabolism, acetyl coenzyme a carboxylase 1, controls T cell immunity. J Immunol. (2014) 192:3190–9. doi: 10.4049/jimmunol.1302985. PMID: 24567531 PMC3965631

[42] Michalek RD Gerriets VA Jacobs SR Macintyre AN MacIver NJ Mason EF . Cutting edge: distinct glycolytic and lipid oxidative metabolic programs are essential for effector and regulatory CD4+ T cell subsets. J Immunol. (2011) 186:3299–303. doi: 10.4049/jimmunol.1003613. PMID: 21317389 PMC3198034

[43] Gerth K Bedorf N Irschik H Höfle G Reichenbach H . The soraphens: a family of novel antifungal compounds from Sorangium cellulosum (Myxobacteria). I. Soraphen A1 alpha: fermentation, isolation, biological properties. J Antibiot (Tokyo). (1994) 47:23–31. doi: 10.1128/9781555815677.ch19 8119858

[44] Shen Y Volrath SL Weatherly SC Elich TD Tong L . A mechanism for the potent inhibition of eukaryotic acetyl-coenzyme A carboxylase by soraphen A, a macrocyclic polyketide natural product. Mol Cell. (2004) 16:881–91. doi: 10.1016/j.molcel.2004.11.034. PMID: 15610732

[45] Vahlensieck HF Pridzun L Reichenbach H Hinnen A . Identification of the yeast ACC1 gene product (acetyl-CoA carboxylase) as the target of the polyketide fungicide soraphen A. Curr Genet. (1994) 25:95–100. doi: 10.1007/bf00309532. PMID: 7916271

[46] Wang X Zhou Y Tang D Zhu Z Li Y Huang T . ACC1 (Acetyl Coenzyme A Carboxylase 1) is a potential immune modulatory target of cerebral ischemic stroke. Stroke. (2019) 50:1869–78. doi: 10.1161/strokeaha.119.024564. PMID: 31177975

[47] Engel O Meisel A . Models of infection before and after stroke: investigating new targets. Infect Disord Drug Targets. (2010) 10:98–104. doi: 10.2174/187152610790963546. PMID: 20166971

[48] Grigoryan R Costas-Rodríguez M Wonterghem EV Vandenbroucke RE Vanhaecke F . Effect of endotoxemia induced by intraperitoneal injection of lipopolysaccharide on the Mg isotopic composition of biofluids and tissues in mice. Front Med. (2021) 8:664666. doi: 10.3389/fmed.2021.664666. PMID: 34368182 PMC8342922

[49] Juskewitch JE Knudsen BE Platt JL Nath KA Knutson KL Brunn GJ . LPS-induced murine systemic inflammation is driven by parenchymal cell activation and exclusively predicted by early MCP-1 plasma levels. Am J Pathol. (2012) 180:32–40. doi: 10.1016/j.ajpath.2011.10.001. PMID: 22067909 PMC3338351

[50] Skrzypczak-Wiercioch A Sałat K . Lipopolysaccharide-induced model of neuroinflammation: mechanisms of action, research application and future directions for its use. Molecules. (2022) 27:5481. doi: 10.3390/molecules27175481. PMID: 36080253 PMC9457753

[51] Jander S Kraemer M Schroeter M Witte OW Stoll G . Lymphocytic infiltration and expression of intercellular adhesion molecule-1 in photochemically induced ischemia of the rat cortex. J Cereb Blood Flow Metab. (1995) 15:42–51. doi: 10.1038/jcbfm.1995.5. PMID: 7528223

[52] Schroeter M Jander S Witte OW Stoll G . Local immune responses in the rat cerebral cortex after middle cerebral artery occlusion. J Neuroimmunol. (1994) 55:195–203. doi: 10.1016/0165-5728(94)90010-8. PMID: 7530260

[53] Schulze J Gellrich J Kirsch M Dressel A Vogelgesang A . Central nervous system-infiltrating T lymphocytes in stroke are activated via their TCR (T-cell receptor) but lack CD25 expression. Stroke. (2021) 52:2939–47. doi: 10.1161/strokeaha.120.032763. PMID: 34266304

[54] Jenkins MK Khoruts A Ingulli E Mueller DL McSorley SJ Reinhardt RL . *In vivo* activation of antigen-specific CD4 T cells. Annu Rev Immunol. (2001) 19:23–45. doi: 10.1146/annurev.immunol.19.1.23 11244029

[55] Liesz A Karcher S Veltkamp R . Spectratype analysis of clonal T cell expansion in murine experimental stroke. J Neuroimmunol. (2013) 257:46–52. doi: 10.1016/j.jneuroim.2013.01.013. PMID: 23498140

[56] Ito M Komai K Mise-Omata S Iizuka-Koga M Noguchi Y Kondo T . Brain regulatory T cells suppress astrogliosis and potentiate neurological recovery. Nature. (2019) 565:246–50. doi: 10.1038/s41586-018-0824-5. PMID: 30602786

[57] Mracsko E Liesz A Stojanovic A Lou WPK Osswald M Zhou W . Antigen dependently activated cluster of differentiation 8-positive T cells cause perforin-mediated neurotoxicity in experimental stroke. J Neurosci. (2014) 34:16784–95. doi: 10.1523/jneurosci.1867-14.2014. PMID: 25505331 PMC6608504

[58] Planas AM Gómez-Choco M Urra X Gorina R Caballero M Chamorro Á . Brain-derived antigens in lymphoid tissue of patients with acute stroke. J Immunol. (2012) 188:2156–63. doi: 10.4049/jimmunol.1102289. PMID: 22287710

[59] Ren X Akiyoshi K Grafe MR Vandenbark AA Hurn PD Herson PS . Myelin specific cells infiltrate MCAO lesions and exacerbate stroke severity. Metab Brain Dis. (2012) 27:7–15. doi: 10.1007/s11011-011-9267-5. PMID: 21989743 PMC3270145

[60] Becker KJ McCarron RM Ruetzler C Laban O Sternberg E Flanders KC . Immunologic tolerance to myelin basic protein decreases stroke size after transient focal cerebral ischemia. Proc Natl Acad Sci USA. (1997) 94:10873–8. doi: 10.1073/pnas.94.20.10873. PMID: 9380727 PMC23514

[61] Frenkel D Huang Z Maron R Koldzic DN Hancock WW Moskowitz MA . Nasal vaccination with myelin oligodendrocyte glycoprotein reduces stroke size by inducing IL-10-producing CD4+ T cells1. J Immunol. (2003) 171:6549–55. doi: 10.4049/jimmunol.171.12.6549. PMID: 14662856

[62] Moran AE Holzapfel KL Xing Y Cunningham NR Maltzman JS Punt J . T cell receptor signal strength in T _reg_ and iNKT cell development demonstrated by a novel fluorescent reporter mouse. J Exp Med. (2011) 208:1279–89. doi: 10.1084/jem.20110308. PMID: 21606508 PMC3173240

[63] Wu J Cai Y Wu X Ying Y Tai Y He M . Transcardiac perfusion of the mouse for brain tissue dissection and fixation. Bio Protoc. (2021) 11:e3988. doi: 10.21769/bioprotoc.3876 PMC8005872 33796622

[64] Pösel C Möller K Boltze J Wagner DC Weise G . Isolation and flow cytometric analysis of immune cells from the ischemic mouse brain. J Vis Exp. (2016) (108):53658. doi: 10.3791/53658 PMC4828148 26967380

[65] Błaż M Natorska J Bembenek JP Członkowska A Ząbczyk M Polak M . Elevated lipopolysaccharide level is largely driven by time since symptom onset in acute ischemic stroke: the impact on clinical outcomes. J Thromb Haemostasis. (2024) 22:3161–71. doi: 1016/j.jtha.2024.06.028 10.1016/j.jtha.2024.06.028 39122194

[66] Doll DN Engler-Chiurazzi EB Lewis SE Hu H Kerr AE Ren X . Lipopolysaccharide exacerbates infarct size and results in worsened post-stroke behavioral outcomes. Behav Brain Funct. (2015) 11:32. doi: 10.1186/s12993-015-0077-5. PMID: 26463864 PMC4604642

[67] Hakoupian M Ferino E Jickling GC Amini H Stamova B Ander BP . Bacterial lipopolysaccharide is associated with stroke. Sci Rep. (2021) 11:6570. doi: 10.1038/s41598-021-86083-8. PMID: 33753837 PMC7985504

[68] Klimiec E Pera J Chrzanowska-Wasko J Golenia A Slowik A Dziedzic T . Plasma endotoxin activity rises during ischemic stroke and is associated with worse short-term outcome. J Neuroimmunol. (2016) 297:76–80. doi: 10.1016/j.jneuroim.2016.05.006. PMID: 27397079

[69] Klimiec E Pasinska P Kowalska K Pera J Slowik A Dziedzic T . The association between plasma endotoxin, endotoxin pathway proteins and outcome after ischemic stroke. Atherosclerosis. (2018) 269:138–43. doi: 10.1016/j.atherosclerosis.2017.12.034. PMID: 29353229

[70] Zhu Y Hu Y Liu Z Chang L Geng X Yin X . The LPS-inactivating enzyme acyloxyacyl hydrolase protects the brain from experimental stroke. Trans Res. (2024) 270:42–51. doi: 10.1016/j.trsl.2024.03.007. PMID: 38522823

[71] Cheung JS Wang X Zhe Sun P . Magnetic resonance characterization of ischemic tissue metabolism. Open Neuroimag J. (2011) 5:66–73. doi: 10.2174/1874440001105010066 PMC3245409 22216079

[72] Turner RC DiPasquale K Logsdon AF Tan Z Naser ZJ Huber JD . The role for infarct volume as a surrogate measure of functional outcome following ischemic stroke. J Syst Integr Neurosci. (2016) 2(4). doi: 10.15761/JSIN.1000136 PMC5347398 28299202

[73] Jia W Kamen Y Pivonkova H Káradóttir RT . Neuronal activity-dependent myelin repair after stroke. Neurosci Lett. (2019) 703:139–44. doi: 10.1016/j.neulet.2019.03.005 30904575

[74] Sims NR Yew WP . Reactive astrogliosis in stroke: Contributions of astrocytes to recovery of neurological function. Neurochem Int. (2017) 107:88–103. doi: 10.1016/j.neuint.2016.12.016 28057555

[75] Schmidt J Reinold J Klinger R Benson S . Systemische Entzündung, „Sickness Behavior“ und Erwartungsprozesse. Schmerz. (2022) 36(3):166–71. doi: 10.1007/s00482-021-00602-0 PMC9156479 34714400

[76] Li P Gan Y Sun BL Zhang F Lu B Gao Y . Adoptive regulatory T-cell therapy protects against cerebral ischemia. Ann Neurol. (2013) 74(3):458–71. doi: 10.1002/ana.23815 PMC3748165 23674483

[77] Stubbe T Ebner F Richter D Engel O Klehmet J Royl G . Regulatory T cells accumulate and proliferate in the ischemic hemisphere for up to 30 days after MCAO. J Cereb Blood Flow Metab. (2013) 33:37–47. doi: 10.1038/jcbfm.2012.149. PMID: 22968321 PMC3597367

[78] Liston A Aloulou M . A fresh look at a neglected regulatory lineage: CD8+Foxp3+ regulatory T cells. Immunol Lett. (2022) 247:22–6. doi: 10.1016/j.imlet.2022.05.004. PMID: 35609830

[79] Zhang L Wei Y Wang D Du J Wang X Li B . Elevated Foxp3+ double-negative T cells are associated with disease progression during HIV infection. Front Immunol. (2022) 13:947647. doi: 10.3389/fimmu.2022.947647. PMID: 35967422 PMC9365964

[80] Chellappa S Hugenschmidt H Hagness M Subramani S Melum E Line PD . CD8+ T cells that coexpress RORγt and T-bet are functionally impaired and expand in patients with distal bile duct cancer. J Immunol. (2017) 198:1729–39. doi: 10.4049/jimmunol.1600061. PMID: 28053236

[81] Guo Y Chen X Li D Liu H Ding Y Han R . PR-957 mediates neuroprotection by inhibiting Th17 differentiation and modulating cytokine production in a mouse model of ischaemic stroke. Clin Exp Immunol. (2018) 193:194–206. doi: 10.1111/cei.13132. PMID: 29603201 PMC6046491

[82] Li Q Ding S Wang YM Xu X Shen Z Fu R . Age-associated alteration in Th17 cell response is related to endothelial cell senescence and atherosclerotic cerebral infarction. Am J Transl Res. (2017) 9:5160–8. PMC5714799 29218113

[83] Mamareli P Kruse F Lu CW Guderian M Floess S Rox K . Targeting cellular fatty acid synthesis limits T helper and innate lymphoid cell function during intestinal inflammation and infection. Mucosal Immunol. (2021) 14:164–76. doi: 10.1038/s41385-020-0285-7. PMID: 32355319

[84] Orlov M Dmyterko V Wurfel MM Mikacenic C . Th17 cells are associated with protection from ventilator associated pneumonia. PLoS One. (2017) 12(8):e0182966. doi: 10.1371/journal.pone.0182966 28806403 PMC5555641

[85] Stanley D Moore RJ Wong CHY . An insight into intestinal mucosal microbiota disruption after stroke. Sci Rep. (2018) 8(1):568. doi: 10.1038/s41598-017-18904-8 29330443 PMC5766598

[86] Wang J Gao Y Yuan Y Wang H Wang Z Zhang X . Th17 cells and IL-17A in ischemic stroke. Mol Neurobiol. (2024) 61(4):2411–29. doi: 10.1007/s12035-023-03723-y PMC10973033 37884768

[87] Way EE Chen K Kolls JK . Dysregulation in lung immunity - the protective and pathologic Th17 response in infection. Eur J Immunol. (2013) 43:3116–24. doi: 10.1002/eji.201343713. PMID: 24130019 PMC3947216

[88] Turner PV . The role of the gut microbiota on animal model reproducibility. Anim Model Exp Med. (2018) 1:109–15. doi: 10.1002/ame2.12022. PMID: 30891555 PMC6388061

[89] Kim CH Park J Kim M . Gut microbiota-derived short-chain fatty acids, T cells, and inflammation. Immune Netw. (2014) 14:277–88. doi: 10.4110/in.2014.14.6.277. PMID: 25550694 PMC4275385

[90] Xie X Wang L Dong S Ge S Zhu T . Immune regulation of the gut-brain axis and lung-brain axis involved in ischemic stroke. Neural Regener Res. (2023) 19:519–28. doi: 10.4103/1673-5374.380869. PMID: 37721279 PMC10581566

[91] Vendrame M Gemma C Pennypacker KR Bickford PC Davis Sanberg C Sanberg PR . Cord blood rescues stroke-induced changes in splenocyte phenotype and function. Exp Neurol. (2006) 199:191–200. doi: 10.1016/j.expneurol.2006.03.017. PMID: 16713598

