# Supplementary material for: Targeting T cell metabolism and polarization to modulate post-stroke immune responses and improve outcomes
Source: Front Immunol. 2026 May 8;17:1703552. doi: 10.3389/fimmu.2026.1703552 (PMC13193879; doi:10.3389/fimmu.2026.1703552)
Supplement: Supplementary file 2 [file DataSheet2.pdf]

Supplemental materials

Supplement S1: Sum Score

Weight loss	<5% weight loss	0
	5-10 % weight loss	1
	11-15 % weight loss	2
	16-20 % weight loss	3
	>20 % weight loss	4
Fur condition	Normal, well-groomed	0
	Slightly unkempt	1
	Slight piloerection	2
	Piloerection, dirty fur, dehydration	3
Behavior /Activity	Normal, lively, curious	0
	Less lively/curious or hyperactive	1
	Nervous/tense during handling, decreased mobility, decreased attention	2
	Distraught during handling, immobility, separation	3
mBS- Neuroscore	No deficits	0
(modified Bederson Score)	Flexion of forelimb	1
	Reduced resistance when nudged from the side, flexion of forelimb without circling	2
	circling	3
	Spinning around its own axis	4/endpoint
Breathing	normal	0
	Slightly increased/labored	1
	Increased/labored, abdominal	2
	weak	3

Supplement S2: Gating strategy of flow cytometric data

Doublets were excluded by plotting forward scatter (FSC)-W against FSC-H, side scatter (SSC)-W against SSC-H and FSC-H against FSC-A (supplemental figure 1A-C). Then,

Zombie+ dead cells were excluded and debris was distinguished from cells by plotting SSC-A against FSC-A (supplemental figure 1D, E). Leukocytes were gated by plotting SSC-A against CD45 and from the CD45+ leukocytes, T cells were distinguished via their expression of CD3. CD4+ and CD8+ T cells as well as double negative T cells (DNT; CD4-CD8-) were distinguished by plotting CD4 against CD8 (supplemental figure 1F-H)

Activation markers CD25, CD69 and PD-1 as well as transcription factor GATA-3 were gated according to fluorescence minus one (FMO) controls (supplemental figure 2). Due to the limited number of cells, FMO gates from the spleen were used for gating activation markers in the other organs as well. The feasibility of this approach was confirmed in comparative experiments during the establishment of staining. The remaining transcription factors T-bet, RORγt and FOXP3 were gated manually by plotting them against SSC-A (supplemental figure 3). The cutoff between GFP+ and GFP- cells was set according to splenocytes from mice that do not express Nur77^{GFP} (supplemental figure 4).

In the brain, gates were set in the ipsilateral sample and then transferred to the corresponding contralateral sample. Due to high background noise, debris was excluded in the brain (SSC-A against Zombie) and blood (SSC-A against CD45) prior to the above-mentioned gating strategy for a more accurate distinction of the single cell population.

Absolute cell counts were calculated using Trucount beads. First, a rough gate was set by plotting SSC-A against FSC-A. From this population beads were distinguished from cells and debris by using the high autofluorescence of the beads in the V-610 and R-670 channels (supplemental figure 5).

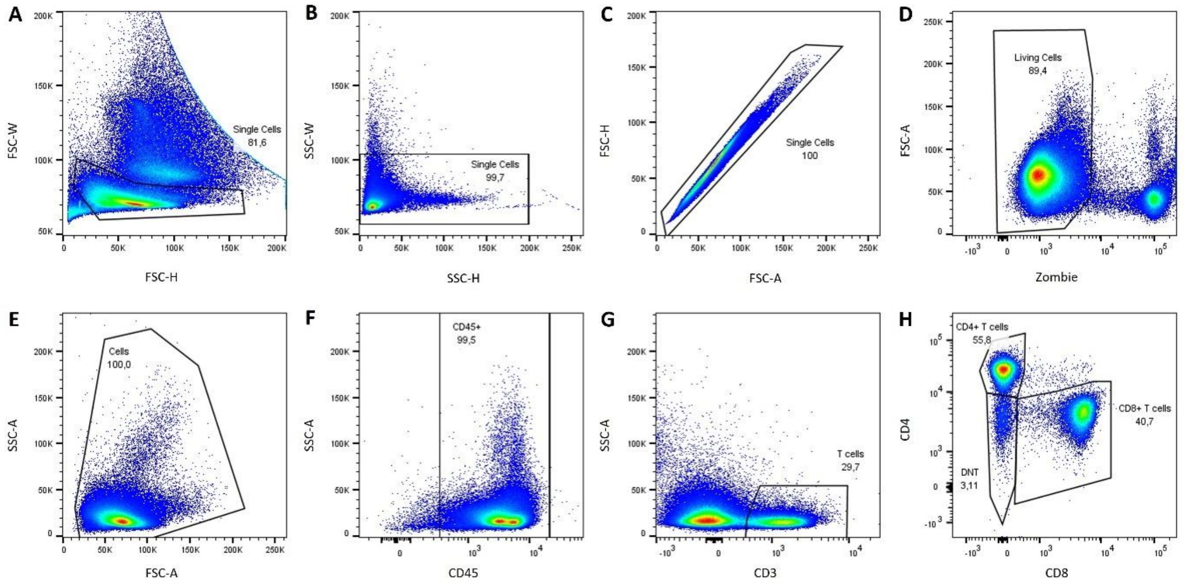

Supplemental figure 1: Exemplary gating strategy of flow cytometry data (FlowJo 10.8.1). (A-C) Doublet exclusion. (D) Dead cell removal. (E) Debris removal. (F) Gating of CD45+ leukocytes. (G) Gating of CD3+T cells. (F) Gating of CD4+, CD8+ and double-negative (CD4-, CD8-)T cells.

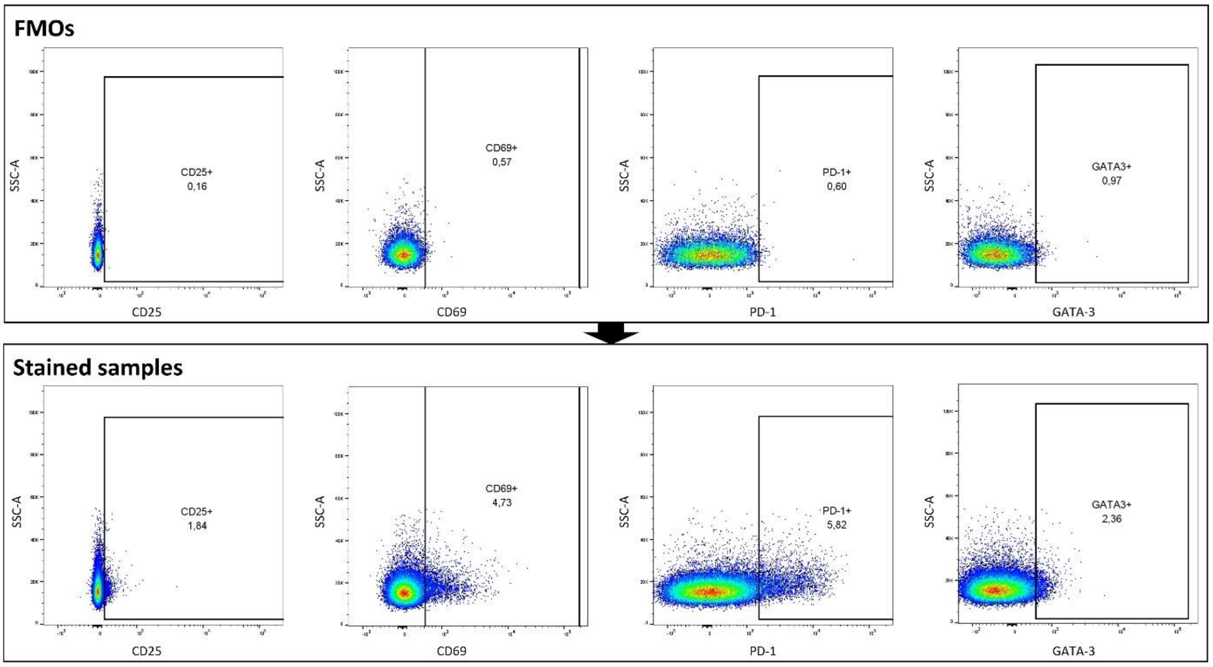

Supplemental figure 2: Gating of activation markers CD25, CD69 and PD-1 as well as transcription factor GATA-3 according to fluorescence minus one (FMO) controls. Due

to the limited number of cells, FMO gates from the spleen were used for gating activation markers in the other organs as well. The feasibility of this approach was confirmed in comparative experiments during the establishment of staining.

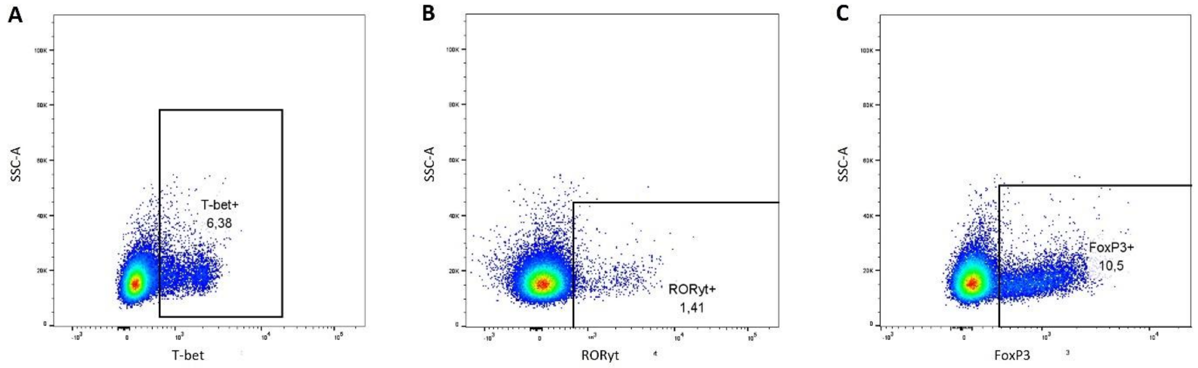

Supplemental figure 3: Manual gating of transcription factors T-bet, RORγt and FoxP3.

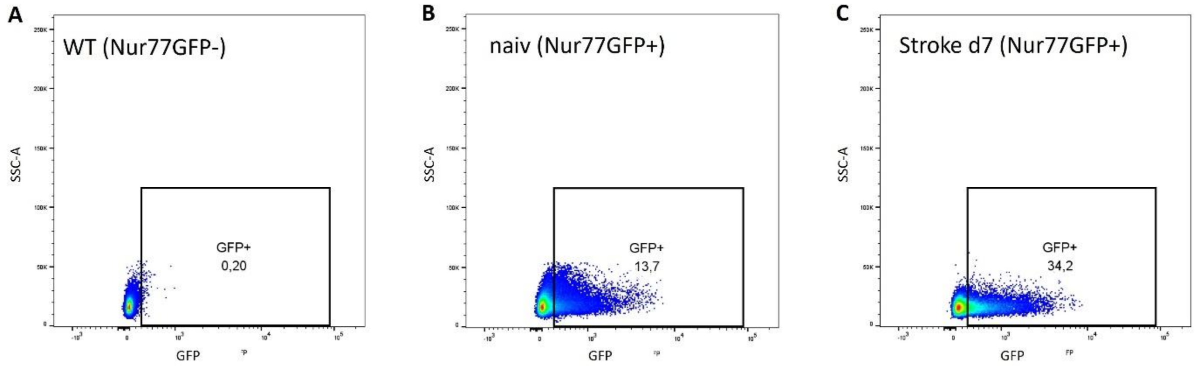

Supplemental figure 4: Gating of GFP+ cells. The cutoff between GFP+ and GFP- cells was set according to splenocytes from mice that do not express $Nur77^{GFP}$ (A).

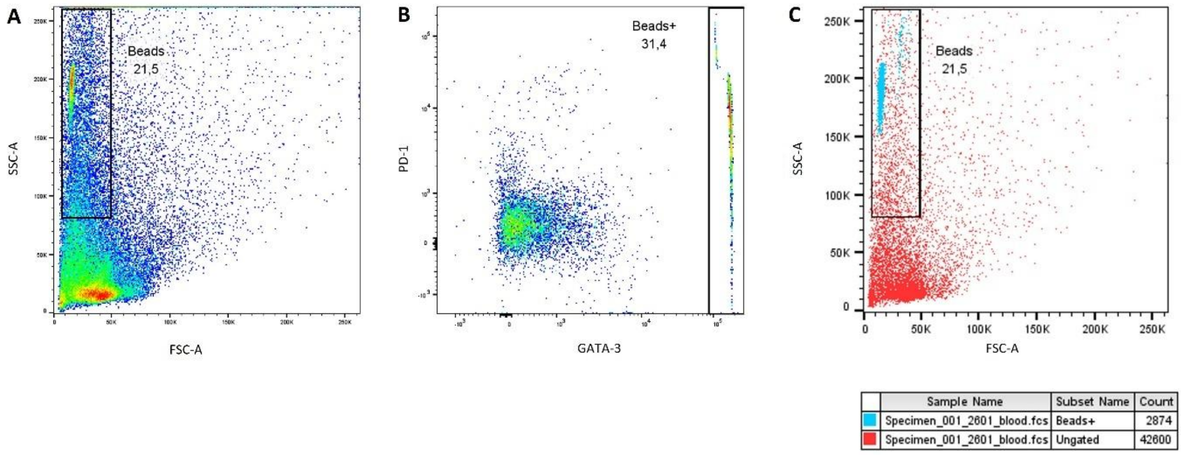

Supplemental figure 5: Gating of Trucount beads for calculation of absolute cell counts.

Supplement S3: …

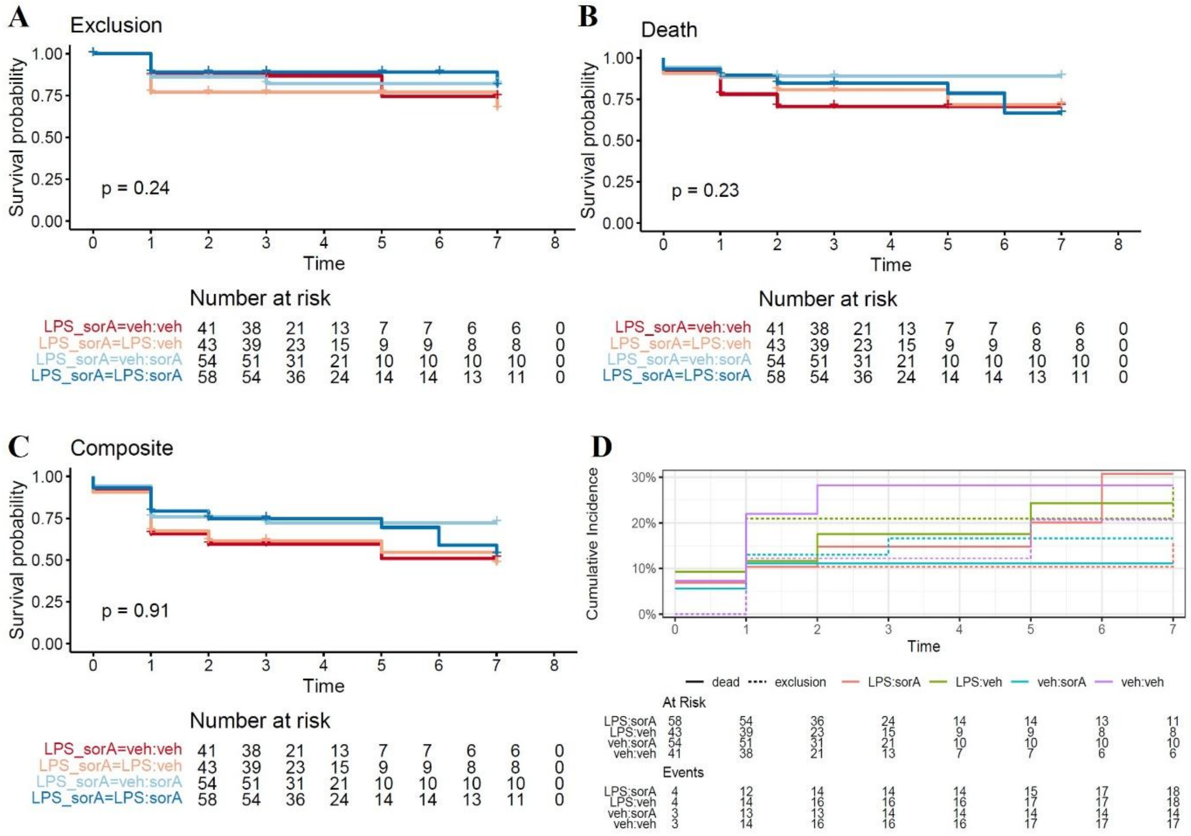

Supplemental figure 6: Kaplan-Meier plots. No treatment effects on death or exclusion rates. We did not detect any differential drop-out between treatment groups for the cause-specific events “exclusion” ($p = 0.24$) (A) “death” ($p = 0.23$) (B), nor for the composite endpoint (death/exclusion; log-rank test: $p = 0.91$) (C). Furthermore, under a competing risk

framework, Gray’s test revealed no differential association of treatment group with the cumulative incidence of “death” ($p = 0.30$) or “exclusion” ($p = 0.50$) (D).

Supplement S4: Additional data

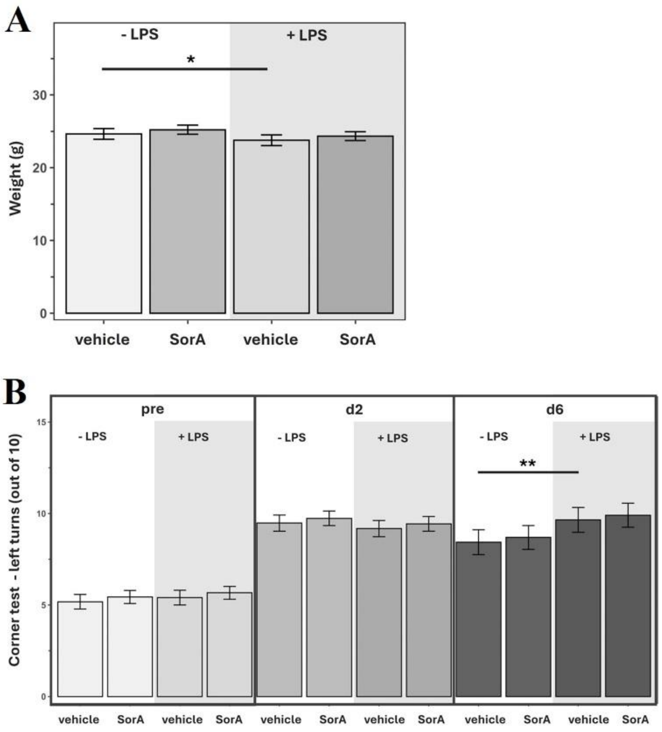

Supplemental figure 7: LPS worsens functional outcome of mice after transient middle cerebral artery occlusion (tMCAO). Data are adjusted for infarct volume and presented as mean ± 95% confidence limits. Linear mixed models were applied. Models included SorA × LPS interaction (2×2 ANOVA); if non-significant, main effect models including only the independent effects of SorA and LPS were applied.

(A) LPS decreases weight after MCAO. Since no interaction with time was detected, effects were averaged across all measured timepoints. *p=0.0250 (-LPS/vehicle: n=24, -LPS/SorA: n=40, +LPS/vehicle: n=24, +LPS/SorA: n=40)

(B) LPS increases number of left turns (out of 10) in the corner test. **p=0.0090 (pre: -LPS/vehicle: n=24, -LPS/SorA: n=40, +LPS/vehicle: n=24, +LPS/SorA: n=40; d2: -LPS/vehicle: n=18, -LPS/SorA: n=30, +LPS/vehicle: n=17,

+LPS/SorA: n=29; d7: -LPS/vehicle: n=6, -LPS/SorA: n=10, +LPS/vehicle: n=6, +LPS/SorA: n=10)

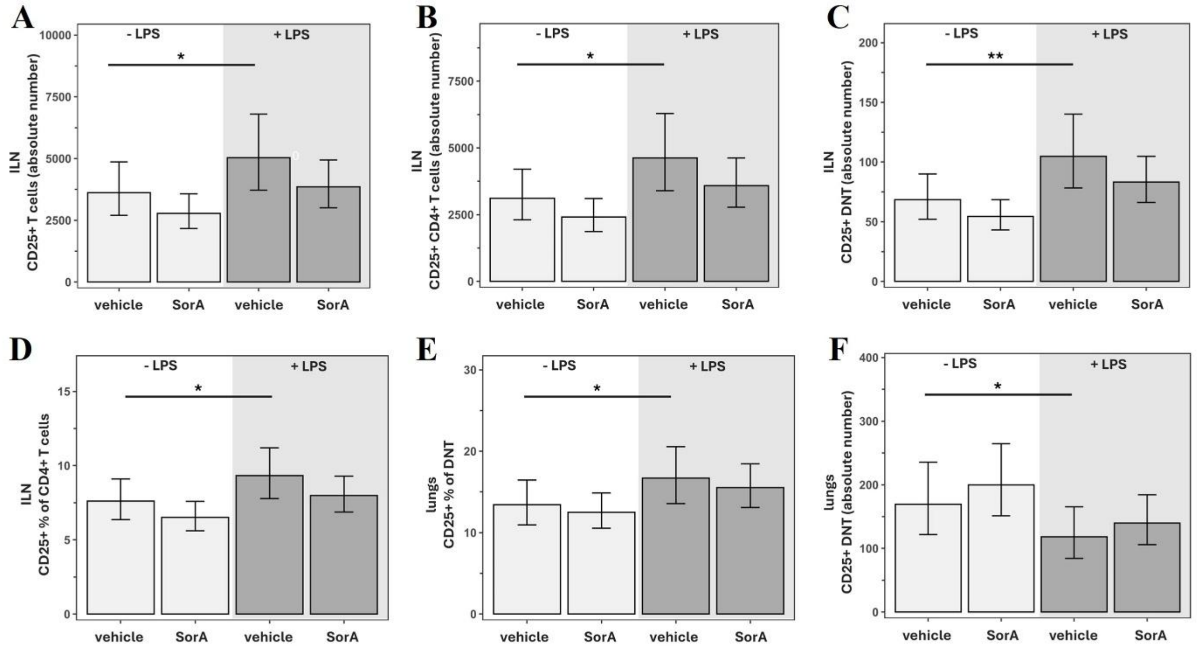

Supplemental figure 8: LPS increases post-MCAO CD25 expression on T cells in inguinal lymph nodes (ILN) and lungs. Data are adjusted for infarct volume and presented as mean ± 95% confidence limits. Since no interaction with time was detected, treatment effects are reported as average effects across all measured timepoints. Linear models / generalized linear models (Gamma-distribution models with a log-link function) were applied and results shown on original scale (back-transformed if needed). Models included SorA × LPS interaction (2×2 ANOVA); if non-significant, main effect models including only the independent effects of SorA and LPS were applied.

(A) Absolute number of CD25+ T cells in the ILN. *p=0.0384
(-LPS/vehicle: n=24, -LPS/SorA: n=40, +LPS/vehicle: n=24, +LPS/SorA: n=40)

(B) Absolute number of CD25+ CD4+ T cells in the ILN. *p=0.0150
(-LPS/vehicle: n=24, -LPS/SorA: n=40, +LPS/vehicle: n=24, +LPS/SorA: n=39)

(C) Absolute number of CD25+ double-negative T cells (CD4-/CD8-, DNT) in the ILN. **p=0.0046
(-LPS/vehicle: n=24, -LPS/SorA: n=40, +LPS/vehicle: n=21, +LPS/SorA: n=39)

(D) Proportion of CD25+ cells within the CD4+ T cell population in the ILN. *p=0.0.0351
(-LPS/vehicle: n=24, -LPS/SorA: n=40, +LPS/vehicle: n=24, +LPS/SorA: n=39)

(E) Proportion of CD25+ cells within double-negative T cells (CD4-/CD8-, DNT) in the lungs. *p=0.0472
(-LPS/vehicle: n=24, -LPS/SorA: n=40, +LPS/vehicle: n=24, +LPS/SorA: n=40)

(F) Absolute number of CD25+ double-negative T cells (CD4-/CD8-, DNT) in the lungs.* p=0.0433
(-LPS/vehicle: n=24, -LPS/SorA: n=40, +LPS/vehicle: n=24, +LPS/SorA: n=40)

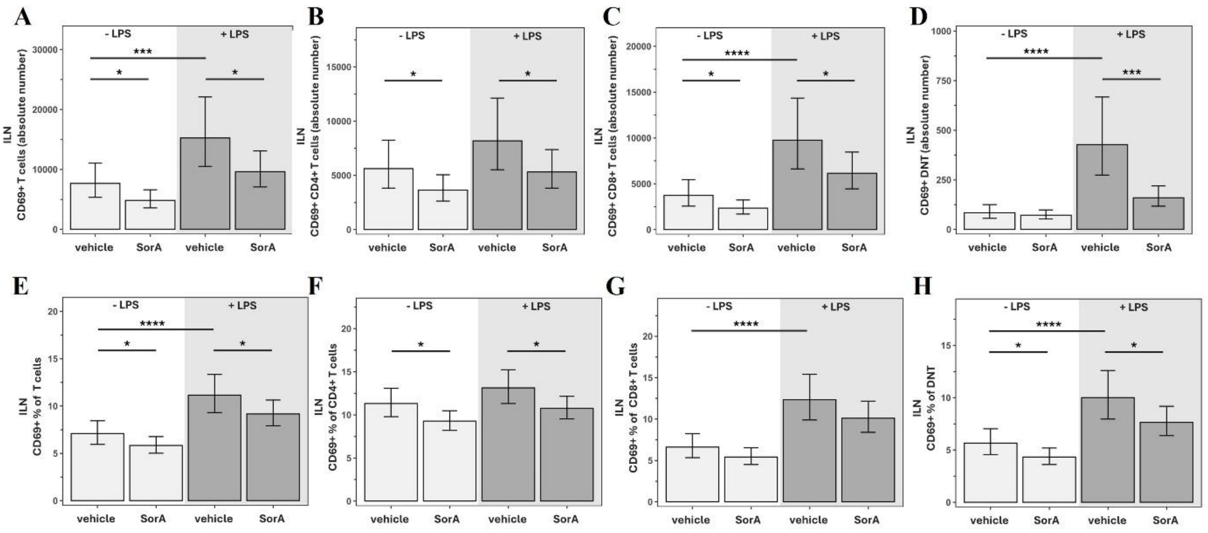

Supplemental figure 9: CD69 expression of T cell populations in the inguinal lymph nodes (ILN) after transient middle cerebral artery occlusion (tMCAO) in mice. Data are adjusted for infarct volume and presented as mean ± 95% confidence limits. Since no

interaction with time was detected, treatment effects are reported as average effects across all measured timepoints. Linear models / generalized linear models (Gamma-distribution models with a log-link function) were applied and results shown on original scale (back-transformed if needed). Models included SorA × LPS interaction (2×2 ANOVA); if non-significant, main effect models including only the independent effects of SorA and LPS were applied.

(A) Absolute number of CD69+ T cells in the ILN. ***p=0.0006, *p=0.0235 (-LPS/vehicle: n=24, -LPS/SorA: n=40, +LPS/vehicle: n=24, +LPS/SorA: n=40)

(B) Absolute number of CD69+ CD4+ T cells in the ILN. *p=0.0442 (-LPS/vehicle: n=24, -LPS/SorA: n=40, +LPS/vehicle: n=24, +LPS/SorA: n=39)

(C) Absolute number of CD69+ CD8+ T cells in the ILN. ****p=0.000006, *p=0.0277 (-LPS/vehicle: n=24, -LPS/SorA: n=40, +LPS/vehicle: n=24, +LPS/SorA: n=39)

(D) Absolute number of CD69+ double-negative T cells (CD4-/CD8-, DNT) in the ILN. ****p=0.0000003, ***p=0.0005 (-LPS/vehicle: n=24, -LPS/SorA: n=40, +LPS/vehicle: n=21, +LPS/SorA: n=39)

(E) Proportion of CD69+ cells within T cells in the ILN. ****p=0.000004, *p=0.0453 (-LPS/vehicle: n=24, -LPS/SorA: n=40, +LPS/vehicle: n=24, +LPS/SorA: n=40)

(F) Proportion of CD69+ cells within CD4+ T cells in the ILN. *p=0.0140 (-LPS/vehicle: n=24, -LPS/SorA: n=40, +LPS/vehicle: n=24, +LPS/SorA: n=39)

(G) Proportion of CD69+ cells within CD8+ T cells in the ILN. ****p=0.0000004 (-LPS/vehicle: n=24, -LPS/SorA: n=40, +LPS/vehicle: n=24, +LPS/SorA: n=39)

(H) Proportion of CD69+ cells within double-negative T cells (CD4-/CD8-, DNT) in the ILN. ****p=0.000003, *p=0.0268 (-LPS/vehicle: n=24, -LPS/SorA: n=40, +LPS/vehicle: n=21, +LPS/SorA: n=39)

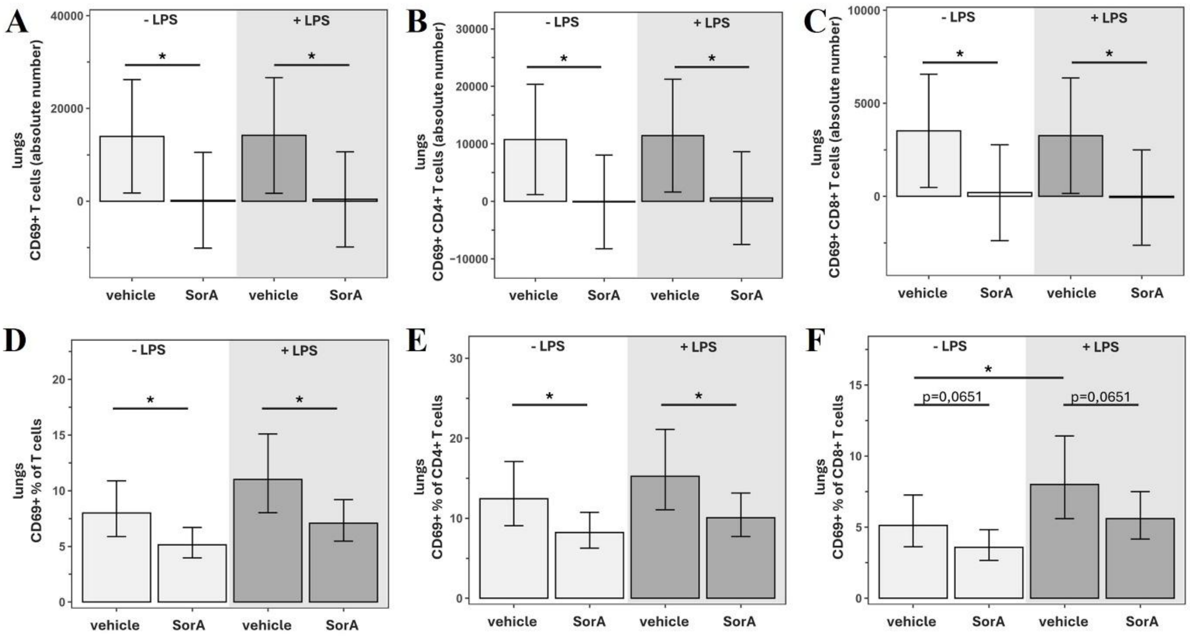

Supplemental figure 10: CD69 expression of T cell populations in the lungs after transient middle cerebral artery occlusion (tMCAO) in mice. Data are adjusted for infarct volume and presented as mean ± 95% confidence limits. Since no interaction with time was detected, treatment effects are reported as average effects across all measured timepoints. Linear models / generalized linear models (Gamma-distribution models with a log-link function) were applied and results shown on original scale (back-transformed if needed). Models included SorA × LPS interaction (2×2 ANOVA); if non-significant, main effect models including only the independent effects of SorA and LPS were applied.

(A) Absolute number of CD69+ T cells in the lungs. *p=0.0424
(-LPS/vehicle: n=24, -LPS/SorA: n=40, +LPS/vehicle: n=24, +LPS/SorA: n=40)

(B) Absolute number of CD69+ CD4+ T cells in the lungs. *p=0.0421
(-LPS/vehicle: n=24, -LPS/SorA: n=40, +LPS/vehicle: n=24, +LPS/SorA: n=40)

(C) Absolute number of CD69+ CD8+ T cells in the lungs. *p=0.0492
(-LPS/vehicle: n=24, -LPS/SorA: n=40, +LPS/vehicle: n=24, +LPS/SorA: n=40)

(D) Proportion of CD69+ cells within T cells in the lungs. *p=0.0107 (-LPS/vehicle: n=24, -LPS/SorA: n=40, +LPS/vehicle: n=24, +LPS/SorA: n=40)

(E) Proportion of CD69+ cells within CD4+ T cells in the lungs. *p=0.0189 (-LPS/vehicle: n=24, -LPS/SorA: n=40, +LPS/vehicle: n=24, +LPS/SorA: n=40)

(F) Proportion of CD69+ cells within CD8+ T cells in the lungs. *p=0.0180 (-LPS/vehicle: n=24, -LPS/SorA: n=40, +LPS/vehicle: n=24, +LPS/SorA: n=40)

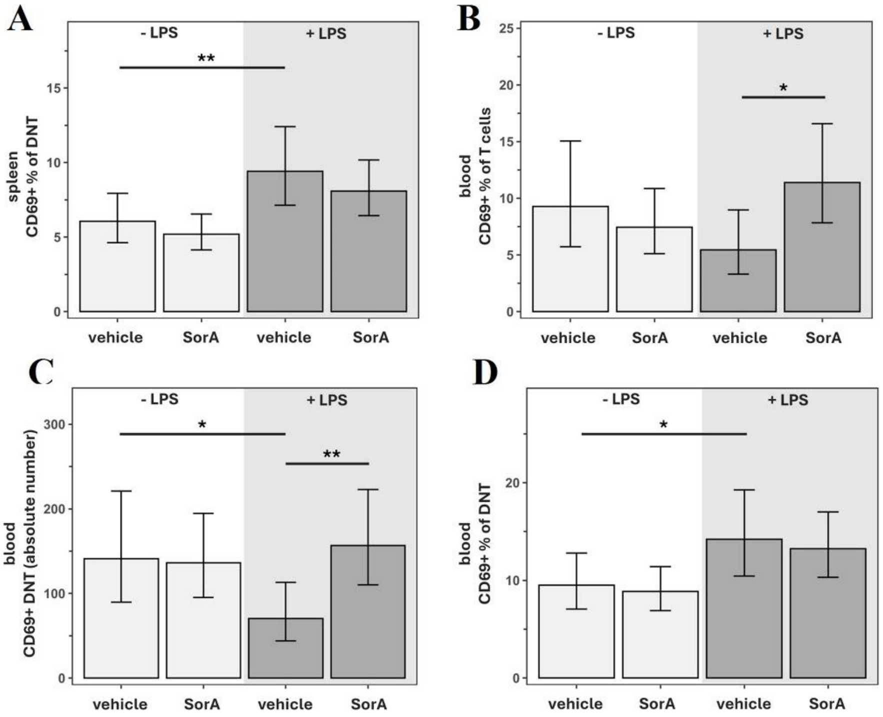

Supplemental figure 11: CD69 expression of T cell populations in the spleen and blood after transient middle cerebral artery occlusion (tMCAO) in mice. Data are adjusted for infarct volume and presented as mean ± 95% confidence limits. Since no interaction with time was detected, treatment effects are reported as average effects across all measured timepoints. Linear models / generalized linear models (Gamma-distribution models with a log-link function) were applied and results shown on original scale (back-transformed if needed). Models included SorA × LPS interaction (2×2 ANOVA); if non-significant, main effect models including only the independent effects of SorA and LPS were applied.

(A) Proportion of CD69+ cells within double-negative T cells (CD4-/CD8-, DNT) in the spleen. **p=0.0027

(-LPS/vehicle: n=24, -LPS/SorA: n=40, +LPS/vehicle: n=24, +LPS/SorA: n=40)

(B) Proportion of CD69+ cells within T cells in the blood. *p=0.0206

(-LPS/vehicle: n=24, -LPS/SorA: n=40, +LPS/vehicle: n=24, +LPS/SorA: n=40)

(C) Absolute number of CD69+ double-negative T cells (CD4-/CD8-, DNT) in the blood. *p=0.0375, **p=0.0084

(-LPS/vehicle: n=24, -LPS/SorA: n=39, +LPS/vehicle: n=23, +LPS/SorA: n=39)

(D) Proportion of CD69+ cells within double-negative T cells (CD4-/CD8-, DNT) in the blood. *p=0.0128

(-LPS/vehicle: n=24, -LPS/SorA: n=39, +LPS/vehicle: n=23, +LPS/SorA: n=39)

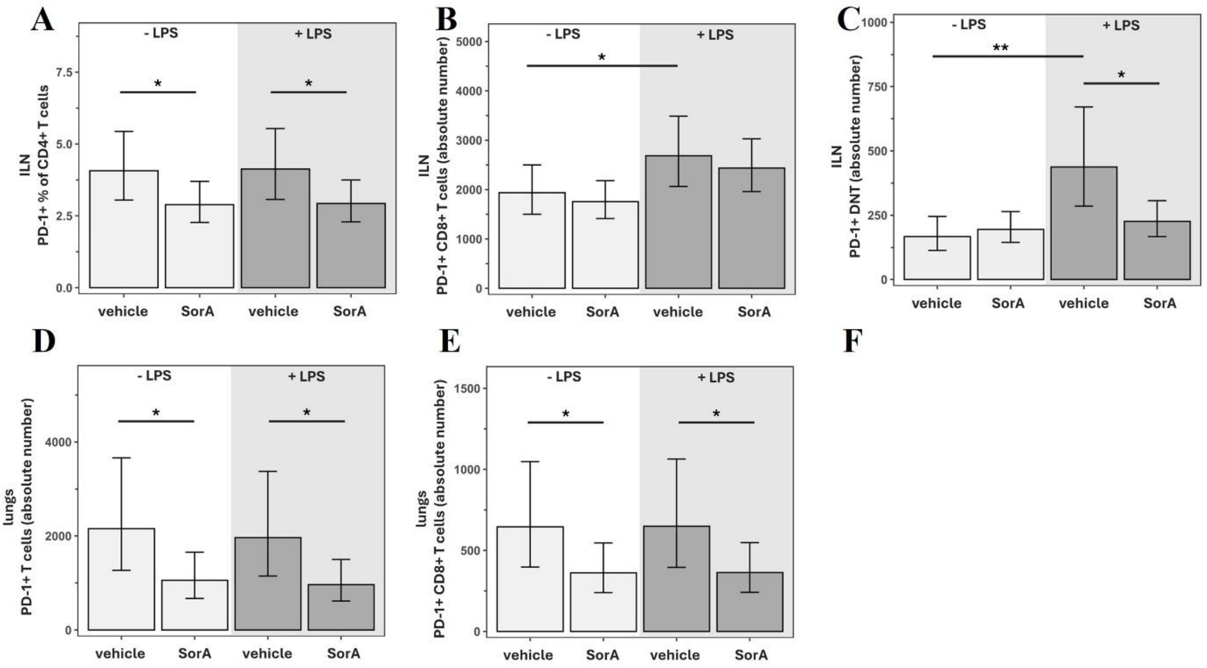

Supplemental figure 12: PD-1 expression of T cell populations in the inguinal lymph nodes (ILN) and lungs after transient middle cerebral artery occlusion (tMCAO) in mice. Data are adjusted for infarct volume and presented as mean ± 95% confidence limits. Since no interaction with time was detected, treatment effects are reported as average effects

across all measured timepoints. Linear models / generalized linear models (Gamma-distribution models with a log-link function) were applied and results shown on original scale (back-transformed if needed). Models included SorA × LPS interaction (2×2 ANOVA); if non-significant, main effect models including only the independent effects of SorA and LPS were applied.

(A) Proportion of PD-1+ cells within T cells in the ILN. *p=0.0338
(-LPS/vehicle: n=24, -LPS/SorA: n=40, +LPS/vehicle: n=24, +LPS/SorA: n=39)

(B) Absolute number of PD-1+ CD8+ T cells in the ILN. *p=0.0186
(-LPS/vehicle: n=24, -LPS/SorA: n=40, +LPS/vehicle: n=24, +LPS/SorA: n=39)

(C) Absolute number of PD-1+ double-negative T cells (CD4-/CD8-, DNT) in the ILN. **p=0.0012, *p=0.0143
(-LPS/vehicle: n=24, -LPS/SorA: n=40, +LPS/vehicle: n=21, +LPS/SorA: n=39)

(D) Absolute number of PD-1+ T cells in the lungs. *p=0.0156
(-LPS/vehicle: n=24, -LPS/SorA: n=40, +LPS/vehicle: n=24, +LPS/SorA: n=40)

(E) Absolute number of PD-1+ CD8+ T cells in the lungs. *p=0.0326
(-LPS/vehicle: n=24, -LPS/SorA: n=40, +LPS/vehicle: n=24, +LPS/SorA: n=40)

(F) Proportion of PD-1+ cells within double-negative T cells (CD4-/CD8-, DNT) in the lungs. **p=0.0064
(-LPS/vehicle: n=24, -LPS/SorA: n=40, +LPS/vehicle: n=24, +LPS/SorA: n=40)

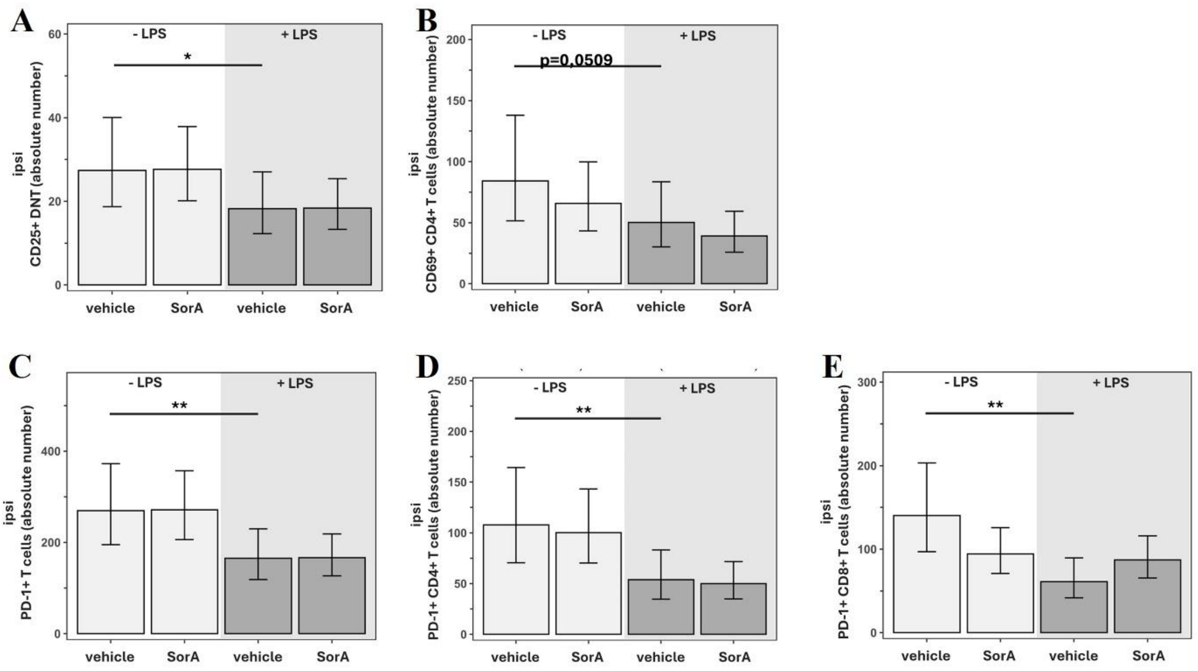

Supplemental figure 13: LPS reduces T cell activation in the ipsilateral hemisphere after transient middle cerebral artery occlusion (tMCAO) in mice. Data are adjusted for infarct volume and presented as mean ± 95% confidence limits. Since no interaction with time was detected, treatment effects are reported as average effects across all measured timepoints. Linear models / generalized linear models (Gamma-distribution models with a log-link function) were applied and results shown on original scale (back-transformed if needed). Models included SorA × LPS interaction (2×2 ANOVA); if non-significant, main effect models including only the independent effects of SorA and LPS were applied.

(A) Absolute number of CD25+ double-negative T cells (CD4-/CD8-, DNT) in the ipsilateral hemisphere. *p=0.0462
(-LPS/vehicle: n=23, -LPS/SorA: n=40, +LPS/vehicle: n=22, +LPS/SorA: n=37)

(B) Absolute number of CD69+ CD4+ T cells in the ipsilateral hemisphere. p=0.0509
(-LPS/vehicle: n=24, -LPS/SorA: n=40, +LPS/vehicle: n=23, +LPS/SorA: n=39)

(C) Absolute number of PD-1+ T cells in the ipsilateral hemisphere. **p=0.0053
(-LPS/vehicle: n=24, -LPS/SorA: n=40, +LPS/vehicle: n=24, +LPS/SorA: n=40)

(D) Absolute number of PD-1+ CD4+ T cells in the ipsilateral hemisphere.

**p=0.0025

(-LPS/vehicle: n=24, -LPS/SorA: n=40, +LPS/vehicle: n=23, +LPS/SorA: n=39)

(E) Absolute number of PD-1+ CD8+ T cells in the ipsilateral hemisphere.

**p=0.0024

(-LPS/vehicle: n=24, -LPS/SorA: n=40, +LPS/vehicle: n=24, +LPS/SorA: n=40)

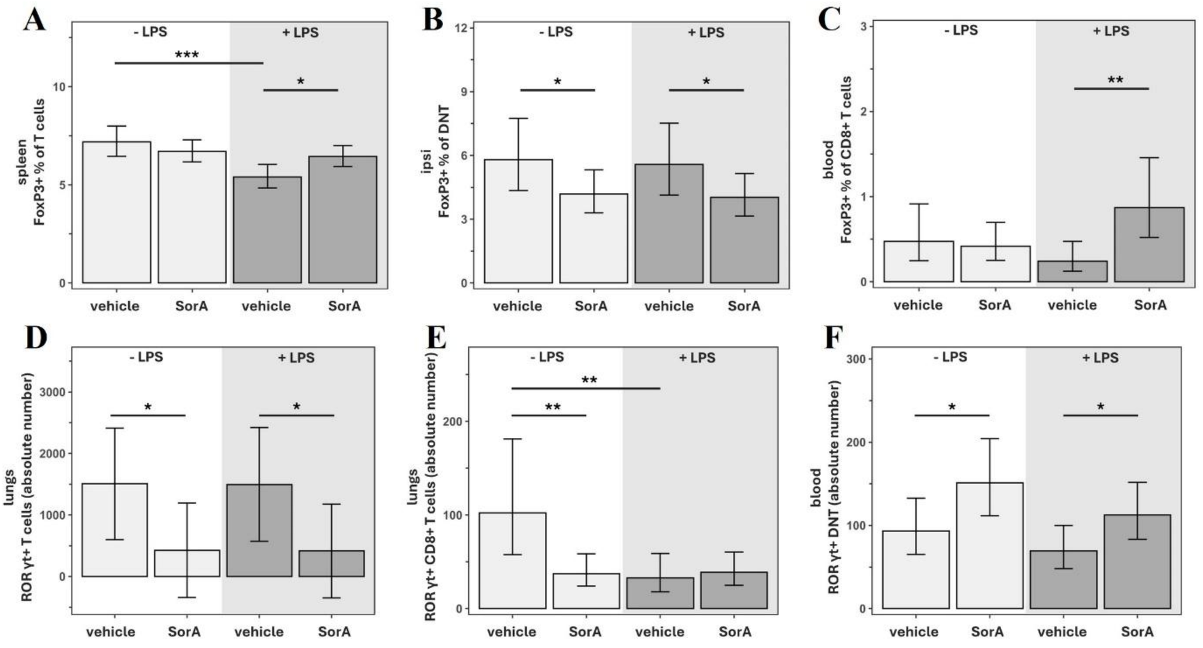

Supplemental figure 14: FoxP3 and RORγt expression after transient middle cerebral artery occlusion (tMCAO) in mice. Data are adjusted for infarct volume and presented as mean ± 95% confidence limits. Since no interaction with time was detected, treatment effects are reported as average effects across all measured timepoints. Linear models / generalized linear models (Gamma-distribution models with a log-link function) were applied and results shown on original scale (back-transformed if needed). Models included SorA × LPS interaction (2×2 ANOVA); if non-significant, main effect models including only the independent effects of SorA and LPS were applied.

(A) Proportion of FoxP3+ cells within T cells in the spleen. ***p=0.0004, *p=0.0131 (-LPS/vehicle: n=24, -LPS/SorA: n=40, +LPS/vehicle: n=24, +LPS/SorA: n=40)

(B) Proportion of FoxP3+ cells within double-negative T cells (CD4-/CD8-, DNT) in the ipsilateral hemisphere. *p=0.0426
(-LPS/vehicle: n=23, -LPS/SorA: n=40, +LPS/vehicle: n=22, +LPS/SorA: n=37)

(C) Proportion of FoxP3+ cells within CD8+ T cells in the blood. **p=0.0034
(-LPS/vehicle: n=24, -LPS/SorA: n=40, +LPS/vehicle: n=24, +LPS/SorA: n=39)

(D) Absolute number of RORγt+ T cells in the lungs. *p=0.0320
(-LPS/vehicle: n=24, -LPS/SorA: n=40, +LPS/vehicle: n=24, +LPS/SorA: n=40)

(E) Absolute number of RORγt+ CD8+ T cells in the lungs. LPS-Effekt **p=0.0070, SorA-Effekt **p=0.0074
(-LPS/vehicle: n=24, -LPS/SorA: n=40, +LPS/vehicle: n=24, +LPS/SorA: n=40)

(F) Absolute number of RORγt+ double-negative T cells (CD4-/CD8-, DNT) in the blood. *p=0.0149
(-LPS/vehicle: n=24, -LPS/SorA: n=39, +LPS/vehicle: n=23, +LPS/SorA: n=39)

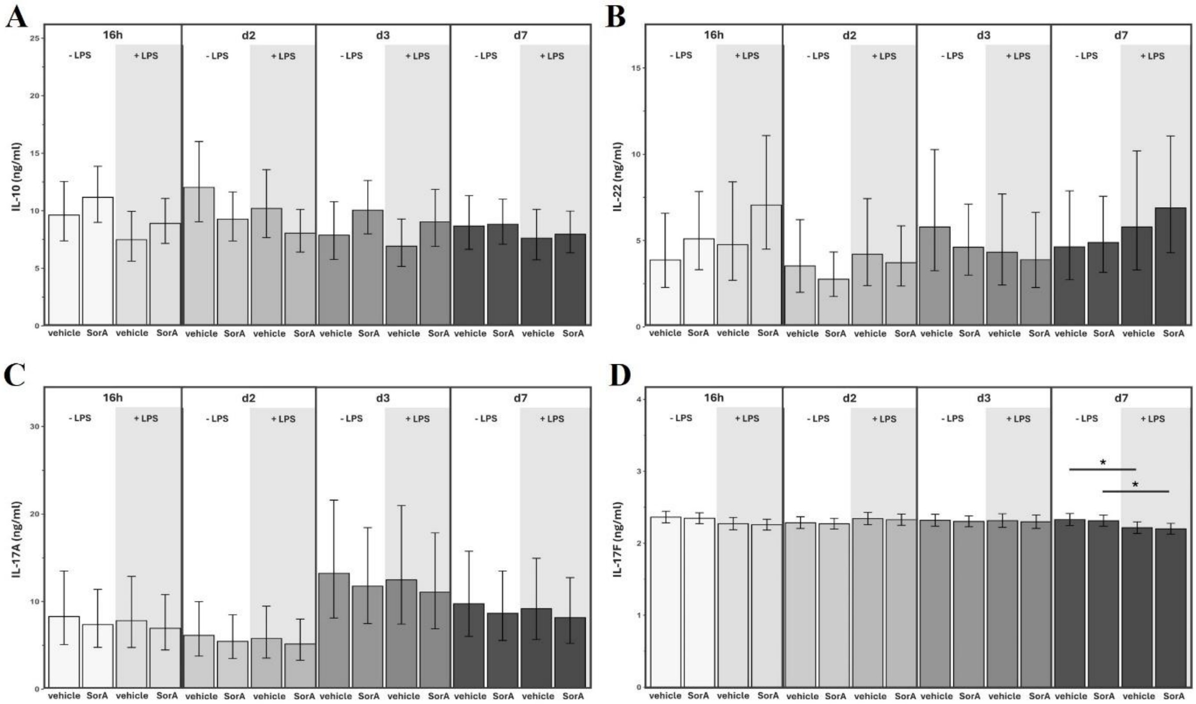

Supplemental figure 15: Soraphen A (SorA) does not change levels of regulatory T cell cytokine IL-10 (A) or Th17 cytokines IL-22 (B), IL-17A (C) and IL-17F (D, *p=0.0362) in the plasma of mice after transient middle cerebral artery occlusion (tMCAO). Data are adjusted for infarct volume and presented as mean ± 95% confidence limits. Linear models / generalized linear models (Gamma-distribution models with a log-link function) were applied and results shown on original scale (back-transformed if needed). Models included SorA × LPS interaction (2×2 ANOVA); if non-significant, main effect models including only the independent effects of SorA and LPS were applied.

(16h: -LPS/vehicle: n=6, -LPS/SorA: n=10, +LPS/vehicle: n=6, +LPS/SorA: n=10; d2:-LPS/vehicle: n=5, -LPS/SorA: n=10, +LPS/vehicle: n=5, +LPS/SorA: n=9; d3: -LPS/vehicle: n=5, -LPS/SorA: n=10, +LPS/vehicle: n=5, +LPS/SorA: n=6; d7: -LPS/vehicle: n=6, -LPS/SorA: n=10, +LPS/vehicle: n=5, +LPS/SorA: n=8)
